# Impact of stromal maturity and proportion on prognosis and immune landscape in colorectal cancer

**DOI:** 10.1080/07853890.2025.2606512

**Published:** 2025-12-26

**Authors:** Vilja V. Tapiainen, Päivi Sirniö, Henna Karjalainen, Ville K. Äijälä, Meeri Kastinen, Vesa-Matti Pohjanen, Hanna Elomaa, Onni Sirkiä, Maarit Ahtiainen, Olli Helminen, Erkki-Ville Wirta, Outi Lindgren, Taneli T. Mattila, Jukka Rintala, Sanna Meriläinen, Juha Saarnio, Tero Rautio, Toni T. Seppälä, Jan Böhm, Jukka-Pekka Mecklin, Anne Tuomisto, Markus J. Mäkinen, Juha P. Väyrynen

**Affiliations:** ^a^ Translational Medicine Research Unit, Medical Research Center Oulu, Oulu University Hospital, and University of Oulu, Oulu, Finland; ^b^ Research Program in Systems Oncology, University of Helsinki, Finland; ^c^ Department of Environmental and Biological Sciences, University of Eastern Finland, Kuopio, Finland; ^d^ Central Finland Biobank, Hospital Nova of Central Finland, Well Being Services County of Central Finland, Jyväskylä, Finland; ^e^ Department of Gastroenterology and Alimentary Tract Surgery, Tampere University Hospital, Tampere, Finland; ^f^ Faculty of Medicine and Health Technology, Tampere University and Tays Cancer Centre, Tampere University Hospital, Tampere, Finland; ^g^ Department of Gastrointestinal Surgery, Helsinki University Central Hospital, University of Helsinki, Helsinki, Finland; ^h^ Applied Tumour Genomics, Research Program Unit, University of Helsinki, Helsinki, Finland; ^i^ Department of Pathology, Hospital Nova of Central Finland, Well Being Services County of Central Finland, Jyväskylä, Finland; ^j^ Department of Education and Research, Well Being Services County of Central Finland, Jyväskylä, Finland; ^k^ Faculty of Sport and Health Sciences, University of Jyväskylä, Jyväskylä, Finland

**Keywords:** Tumour-stroma ratio, desmoplastic reaction, SMAPS, Alcian blue stain, immune cells, colorectal cancer

## Abstract

**Background:**

Tumour microenvironment and cancer cells have constant interaction affecting cancer progression. Tumour-stroma ratio (TSR) in the tumour centre and desmoplastic reaction (DR) classification at the invasive margin are prognostic factors based on stroma evaluation on H&E slides. However, their combined value and immunological associations remain poorly defined. This study examines the prognostic and immunological value of TSR, DR, and their combination in two large colorectal cancer cohorts.

**Methods:**

Two colorectal cancer cohorts (*N* = 1,876) were analyzed. We introduced a three-tiered Stromal Maturity and Proportion Score (SMAPS) based on the presence of high (>50%) TSR and myxoid stroma (immature DR classification). Alcian blue staining was used to further quantify myxoid stroma. Multiplex immunohistochemistry combined with digital image analyses, was utilized to study immune cell densities associated with SMAPS, TSR, DR, and Alcian blue intensity.

**Results:**

In the study cohort (*N* = 1,100), SMAPS was a stronger predictor of cancer-specific mortality [HR for high (vs. low) SMAPS 2.01 (95% CI 1.47–2.75), *p* < 0.0001] compared to TSR [HR for stroma-high (vs. stroma-low) 1.49 (95% CI 1.15–1.93), *p* = 0.003] and DR classification [HR for immature (vs. mature) 1.84 (95% CI 1.39–2.45), *p* < 0.0001]. High SMAPS, stroma-high TSR, and immature DR correlated with lower densities of CD3^+^ T cells, B cells, M1-like macrophages, CD66B^+^ granulocytes, and mast cells. Alcian blue staining was associated with immature DR and corresponding immune cells. The validation cohort (*N* = 776) confirmed the association of SMAPS with survival and T cell densities.

**Conclusions:**

TSR and DR are independent prognostic factors for cancer-specific survival. SMAPS is a promising prognostic tool that integrates stromal maturity at the invasive margin and stromal proportion in the tumour centre. SMAPS has stronger prognostic value compared to TSR and DR classifications alone. A high stromal proportion and myxoid content are associated with an immunosuppressive microenvironment characterized by lower densities of antitumourigenic immune cells.

## KEY MESSAGES

Stromal Maturity and Proportion Score (SMAPS), integrating desmoplastic reaction (DR) classification at the invasive margin and tumour-stroma ratio (TSR) in the tumour centre, demonstrated stronger prognostic value compared to DR and TSR classification alone. Stroma-high TSR, myxoid stroma, and high SMAPS were associated with an immunosuppressive microenvironment characterized by lower densities of antitumourigenic immune cells. Alcian blue stain was associated with myxoid stroma and corresponding immune cell findings.

## Introduction

The dynamic interaction between cancer cells and the tumour microenvironment affects cancer progression [1]. The tumour microenvironment comprises both noncellular and cellular components [2,3]. The noncellular component is dominated by the extracellular matrix (ECM) that contains structural and regulatory proteins, such as collagen and fibronectin [2]. The main stromal cell populations include immune cells, cancer-associated fibroblasts (CAFs), endothelial cells, and adipocytes [2,3]. Together, these stromal components contribute to cancer progression by remodelling the ECM, promoting cell migration, and secreting regulatory factors [4,5]. In turn, cancer cells also actively modify the stromal compartment by altering ECM composition and structure [4].

Histomorphological parameters assessing tumour-induced stromal changes have the potential to refine prognostic evaluation in colorectal cancer (CRC) [6–10]; however, they are not yet routinely implemented in clinical practice. Tumour-stroma ratio (TSR) and desmoplastic reaction (DR) classification are promising prognostic factors based on stromal evaluation from Haematoxylin-eosin (H&E)-stained slides [10,11]. CAFs are important players promoting desmoplastic reaction through their roles in ECM production and remodelling [12]. Because these prognostic factors do not require additional tests or equipment, they can be easily implemented for almost all patients with CRC. However, their combined prognostic utility and association with the detailed composition of the tumour microenvironment remain insufficiently characterized.

TSR is visually evaluated from the tumour centre, estimating stromal content in 10% increments under a x10 objective hotspot containing the most stroma [11]. Tumours are categorized as stroma-high (>50%) and stroma-low (≤50%) [11], with the stroma-high phenotype linked to adverse outcomes in colon cancer [11,13–15], other gastrointestinal malignancies [16–18], and breast cancer [19,20].

DR classification evaluates the invasive margin based on the presence of myxoid stroma and keloid-like collagen [10]. Myxoid stroma appears as basophilic or amphophilic mucinous substance, while keloid-like collagen consists of thick bundles of eosinophilic hyalinized collagen [10]. DR is classified into immature, intermediate, and mature types [10], with the immature category most strongly associated with poor prognosis and reduced densities of tumour-infiltrating memory cytotoxic T cells and stromal M1-like macrophages [8,21–24].

This study aims to refine stromal assessment by introducing the Stromal Maturity and Proportion Score (SMAPS), integrating TSR and DR classifications. We study the prognostic and immunological significance of SMAPS, TSR, and DR in two large CRC cohorts. Additionally, we investigate the use of Alcian blue staining to quantify myxoid stroma, potentially offering a more standardized approach for evaluating stromal maturity.

## Methods

### Study population

The study comprises two cohorts of surgically treated stage I-IV colorectal cancer patients. The primary study cohort was retrospectively collected from Central Finland Central Hospital and includes patients who underwent surgery between 2000 and 2015 (*N* = 1,343). The validation cohort was prospectively gathered from Oulu University Hospital, consisting of patients operated on between 2006 and 2020 (*N* = 1,011). Patients who had received preoperative radiotherapy or chemoradiotherapy (study cohort, *N* = 243; validation cohort, *N* = 235), were excluded to minimize the impact of treatment-induced morphological changes. This resulted in a final study population of 1,100 patients in the study cohort and 776 in validation cohort. Additionally, patients, who had died within 30 days of surgery (study cohort, *N* = 34; validation cohort, *N* = 5) were excluded from survival analyses, resulting in 1,063 patients in the study cohort and 771 patients in the validation cohort. The patient flow is illustrated in Figure S1.

The study endpoints were colorectal cancer-specific survival (CSS) (time from operation to CRC death or end of follow-up) and overall survival (time from operation to death or end of follow-up). There were 296 CRC deaths of 531 deaths in the study cohort and 155 CRC deaths of 284 deaths in the validation cohort during the follow-up. Follow-up was limited to 10 years, because most CRC deaths occur within this period. The median follow-up time for censored cases was 10 years (IQR 7.3–10) in the study cohort and 7.0 years (IQR 4.7–10) in the validation cohort.

### Histopathologic analysis

H&E-stained samples were digitized with Hamamatsu (NanoZoomer S60 or NanoZoomer-XR) or Leica Aperio AT2 slide scanners and assessed *via* digital microscopy (NDP.view2 or Aperio ImageScope). Basic tumour and patient characteristics were previously collected [25–27]. Tumour budding was evaluated according to ITBCC guidelines [28]. Tumour grade and growth patterns were assessed following WHO 2019 criteria. SARIFA was defined as the direct contact between a tumour cell cluster and adipocytes at the invasive margin and was evaluated as described before [29]. Mismatch repair (MMR) status and *BRAF* V600E mutation status were determined using immunohistochemistry.

**Figure 1.**
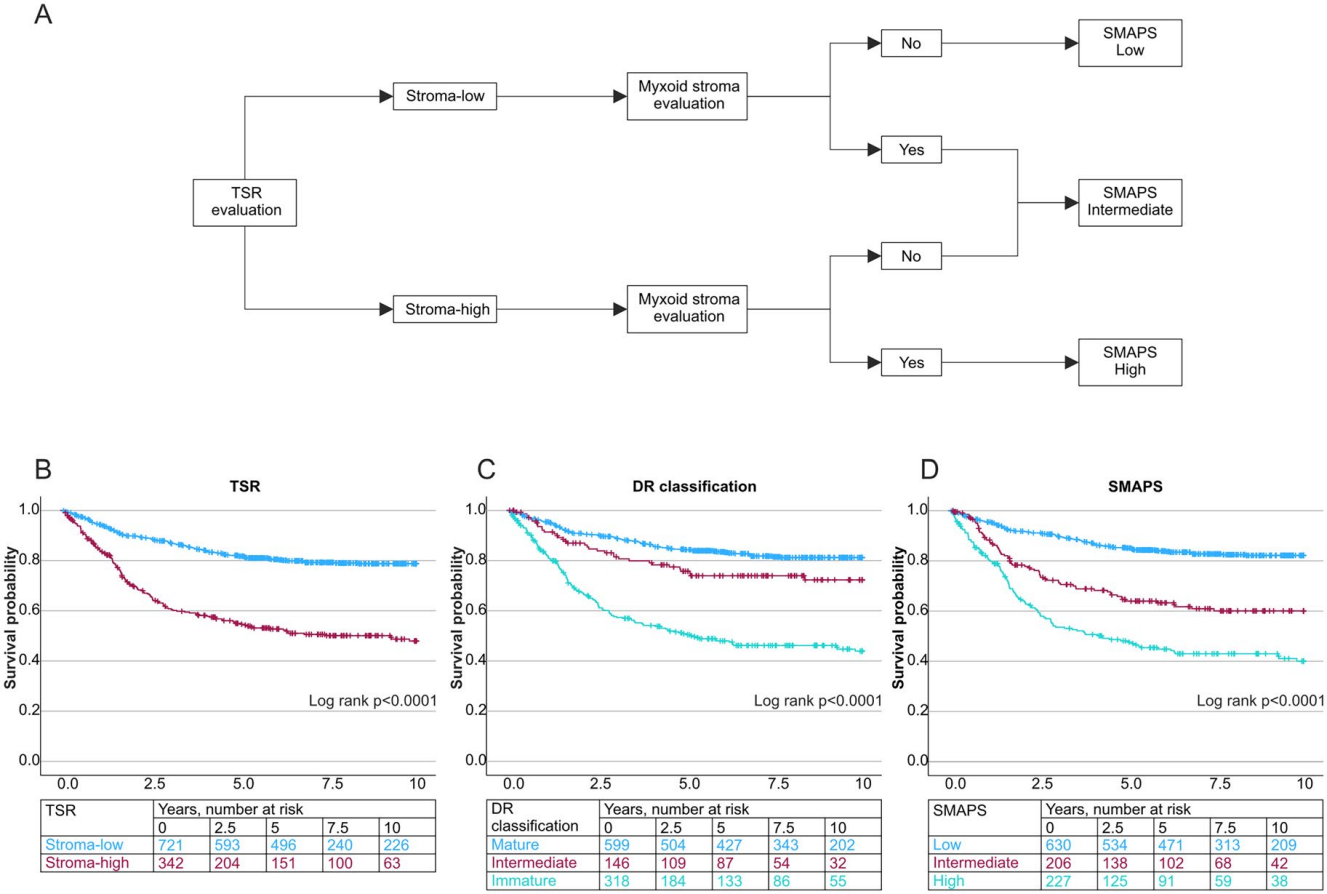
Stromal Maturity and Proportion Score (SMAPS). (A) A flowchart of SMAPS determination based on tumour-stroma ratio (TSR) and myxoid stroma evaluations. Kaplan-Meier curves for cancer-specific survival according to TSR (B), DR classification (C), and SMAPS (D). Abbreviations: TSR: tumour-stroma ratio; DR: desmoplastic reaction; SMAPS: Stromal Maturity and Proportion Score.

**Figure 2.**
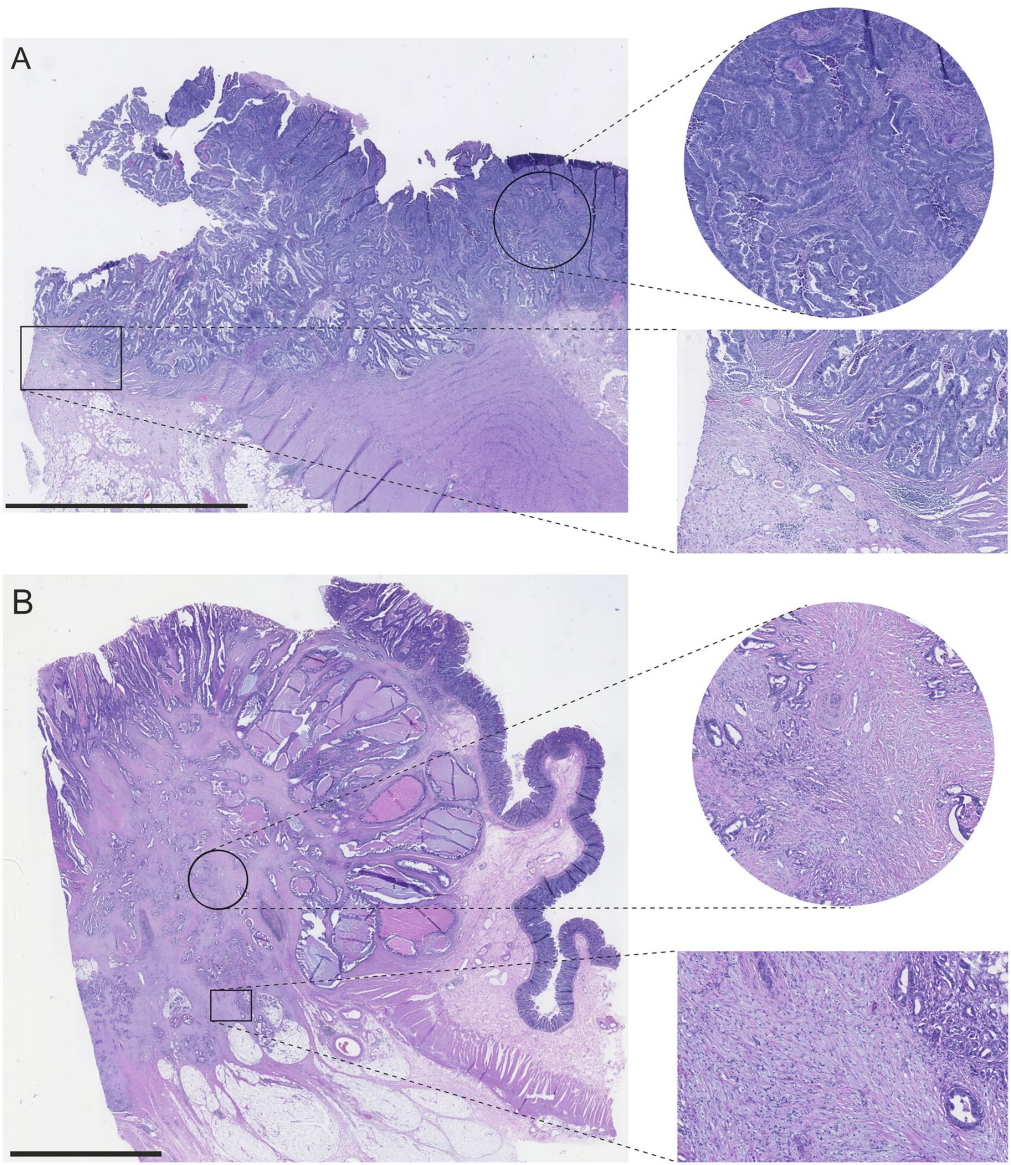
Example images of tumours with low and high Stromal Maturity and Proportion Score (SMAPS). SMAPS low (A) is characterized by stroma-low tumour-stroma ratio and mature stroma. SMAPS high (B) is identified by stroma-high tumour-stroma ratio and myxoid stroma. Scale bars are 5.0 mm.

TSR was visually assessed from H&E-stained sections using a 2-mm-diameter hotspot containing the highest stromal content, as per established criteria [11]. Stromal content was estimated in 10% increments, and tumours were categorized into stroma-high (>50%) and stroma-low (≤50%) [11]. No additional cut-points were screened for this study. For specific CRC morphologic subtypes such as mucinous and medullary carcinomas, stromal evaluations followed the same principles as for other tumour types. Following previously defined criteria [11], extracellular mucus, tumour necrosis, smooth muscle, glandular lumens, and large vessels were excluded from the field of view when evaluating TSR, whereas areas of abundant immune cell infiltration were included in the scoring. DR was evaluated using Ueno’s classification, categorizing cases into immature, intermediate, and mature based on the presence of myxoid stroma and keloid-like collagen [10]. The presence of myxoid stroma indicated immature stroma regardless of keloid-like collagen [10]. Cases with keloid-like collagen without myxoid stroma belonged to the intermediate group [10]. The mature group contained cases without myxoid stroma or keloid-like collagen [10]. The histopathologic assessment of DR and TSR was performed by a gastrointestinal pathologist JPV in the study cohort and researcher VVT in the validation cohort. Training sessions between JPV and VVT were conducted before the evaluation. To investigate interobserver agreement, VVT analyzed 30 consecutive cases from the study cohort blinded to the original evaluations. In the reproducibility analyses, Cohen’s kappa was 0.69 for TSR, 0.79 for DR and 0.67 for SMAPS, representing substantial agreement. Example images of discrepant cases regarding SMAPS evaluation are presented in Figure S2. In the study cohort, the evaluations were based on a single whole-slide image per case, representing the deepest invasion. In the validation cohort, an average of 3 (range 1–18) whole-slide images were evaluated per case. Specifically, all available tumour slides were reviewed for patients operated between 2006 and 2013, whereas for patients operated from 2014 onwards, three representative slides, including the slide with deepest invasion, were evaluated. To assess the impact of slide sampling, 30 consecutive cases from validation cohort were re-evaluated. TSR, DR, and SMAPS scored on a single slide containing the deepest invasion were compared with scores derived from multiple slides. Cohen’s kappa values for single-slide vs. multi-slide assessment were 0.73 for TSR, 0.90 for DR, and 0.80 for SMAPS. The classification based on the deepest invasion slide agreed with the multi-slide assessment in 26/30 cases (87%) for TSR, 28/30 cases (93%) for DR, and 26/30 cases (87%) for SMAPS. The SMAPS was determined based on the evaluations of TSR and DR (Figure 1). Example images of SMAPS low (stroma-low TSR & mature stroma) and SMAPS high (stroma-high TSR & immature stroma) cases are presented in Figure 2. All assessments were performed blinded to study endpoints.

### Immune cell analyses and Alcian blue staining

Tissue microarrays consisting of 1-mm-diameter tumour cores (aiming for 2 tumour centre cores and 2 invasive margin cores per patient) were used to assess immune cell densities and Alcian blue staining intensities in the study cohort. Multiplex immunohistochemistry was employed to evaluate immune cell infiltration in the study cohort. The immune cell quantification data were derived from previously published analyses [30–32]. The associations of immune cell data with TSR, DR classification and SMAPS were analyzed for this study and have not been reported before. The multiplex protocol utilized a cyclic staining method with AEC as the chromogen. A total of three assays were included in this study, with the protocols and antibodies provided in Figure S3. Immune cell quantification was performed using QuPath [33]. Tumour epithelial and stromal regions were identified using random forest pixel classifiers, and various cell populations were detected using random forest object classifiers [30–32]. The analyzed immune cell populations included CD3^+^ T cells, CD20^+^CD79A^+^ B cells, CD20^-^CD79A^+^ plasma cells, CD68^+^/CD163^+^ macrophages, M1-like and M2-like macrophages [categorized based on a polarization index (CD86^+^HLA-DR)-(CD163^+^MRC1), with marker names denoting intensity percentiles across all cases], CD14^+^ monocytic cells, CD14^+^HLA-DR^+^ mature monocytic cells, CD14^+^HLA-DR^-^ immature monocytic cells, CD66B^+^ granulocytes and tryptase^+^ mast cells. Additionally, in both cohorts, conventional immunohistochemistry was applied to quantify CD3^+^ T cell and CD8^+^ T cells, enabling the derivation of the immune cell score based on Immunoscore^®^ principles [27,34].

To quantify myxoid stroma, Alcian blue staining, coupled with cytokeratin immunohistochemistry, was performed. Tissue microarrays were stained for cytokeratin (Leica AE1/AE3, 1:100) using Leica BOND RX stainer, with BOND Epitope Retrieval Solution 2 (Leica, AR9640) pretreatment, followed by detection with the BOND Polymer Refine Detection kit (Leica, DS9800). The standard haematoxylin counterstain was replaced with Alcian blue (Acros 33864-99-2) to stain acidic polysaccharides. In QuPath (v0.4.3.), stain vectors were optimized to detect Alcian blue (blue) and diaminobenzidine (brown). A random forest pixel classifier was trained to distinguish tumour epithelial and stromal regions, while excluding mucus, necrosis, white space, and artefacts such as tissue folds. The mean intensity of Alcian blue staining within stromal areas was measured using the ‘Add Intensity Features’ function. These steps were automated and applied to all images. For statistical analysis, the average Alcian blue intensity in the invasive margin cores was used for each tumour. Alcian blue staining intensity was dichotomized using a cutoff of 0.03 intensity units, defining low versus high intensity. This threshold was derived from the distribution (histogram) of Alcian blue values and was chosen to yield a proportion of cases comparable to those classified as having immature DR on H&E staining.

### Statistical analysis

Statistical analyses were performed using IBM SPSS Statistics for Windows (IBM Corp. version 29.0). Relationships of tumour and patient characteristics with the stromal features (TSR, DR, and SMAPS) were assessed using cross-tabulation and the Chi-square test. Immune cell densities were visualized as boxplots, with statistical differences evaluated using the Mann-Whitney *U* test or Kruskal-Wallis test. The impact of stromal features on cancer-specific and overall survival was studied using Kaplan-Meier survival curves and Cox proportional hazards regression models. For multivariable Cox regression models, the following covariates were included: age (<65, 65–75, >75), sex (male, female), year of operation (Study cohort: 2000–2005, 2006–2010, 2011–2015; Validation cohort: 2006–2010, 2011–2015, 2016–2020), tumour location (proximal colon, distal colon, rectum), AJCC disease stage (I-II, III, IV), tumour grade (low-grade, high-grade), lymphovascular invasion (no, yes), tumour budding (Bd1, Bd2, Bd3), MMR status (proficient, deficient), and *BRAF* mutation status (wild-type, mutant). To minimize degrees of freedom in multivariable models, cases with missing *BRAF* mutation status (2 cases in the study cohort 1, 7 cases in the validation cohort) were categorized as wild-type (the majority category). Receiver operating characteristics (ROC) analysis for cancer-specific survival was performed for the study cohort to compare the prognostic discrimination of SMAPS with TSR, DR classification, tumour budding, and SARIFA. Areas under the ROC curve (AUCs) with 95% confidence intervals were calculated. A subgroup analysis of stage II patients in the study cohort was performed to assess the value of stromal features and tumour budding in identifying high-risk stage II patients. The multivariable Cox regression models for subgroup analysis were adjusted for the aforementioned covariates, excluding disease stage and tumour budding. The potential interaction between SMAPS and adjuvant therapy was evaluated in the validation cohort using Kaplan-Meier analyses and an interaction term in Cox regression models_._ P_interaction_ was calculated using the Wald test for the cross product of SMAPS (low vs. intermediate & high) and adjuvant therapy (no vs. yes) in the Cox regression model. In all analyses, *p*-values of <0.005 were considered statistically significant based on the recommendation of an expert panel, aimed at reducing the risk of type 1 error [35].

## Results

### Associations of TSR and DR with patient and tumour characteristics

We first examined the relationship of TSR and DR with patient and tumour characteristics for 1,100 patients in the primary study cohort. For TSR, the majority of patients (68%) were classified as stroma-low, while 357 (32%) were stroma-high (Table S1). High TSR was significantly associated with advanced disease stage (*p* < 0.0001), high tumour grade (*p* < 0.001), micropapillary growth pattern (*p* < 0.0001), presence of lymphovascular invasion (*p* < 0.0001), higher tumour budding grade (*p* < 0.0001), SARIFA positivity (*p* < 0.0001), and MMR proficient status (*p* < 0.0001). In DR classification, 613 (56%) patients had a mature stromal response, 154 (14%) were classified as intermediate, and 333 (30%) had an immature DR pattern (Table S2). Immature DR was significantly associated with colon tumour location (*p* = 0.003), advanced disease stage (*p* < 0.0001), high tumour grade (*p* < 0.0001), micropapillary growth pattern (*p* < 0.0001), presence of lymphovascular invasion (*p* < 0.0001), higher tumour budding grade (*p* < 0.0001), SARIFA positivity (*p* < 0.0001), and MMR proficient status (*p* = 0.003).

### Prognostic impact of TSR and DR

In Kaplan-Meier analyses, the stroma-high TSR group was associated with shorter 10-year cancer-specific survival than the stroma-low group (Log rank *p* < 0.0001) (Figure 1). In DR classification, patients with an immature DR had shorter 10-year survival than those in the intermediate or mature groups (Log rank *p* < 0.0001) (Figure 1).

In univariable and multivariable Cox regression model analyses, TSR and DR classification were significantly associated with cancer-specific mortality (Table 2, Table S3). Multivariable HR for CRC death in the stroma-high (vs. stroma-low) TSR group was 1.49 (95% CI: 1.15–1.93, *p* = 0.003). The multivariable HRs for CRC death in intermediate and immature DR groups (vs. mature) were 1.11 (95% CI: 0.75–1.66) and 1.84 (95% CI: 1.39–2.45, *P*_trend_<0.0001), respectively. These findings highlight the independent prognostic value of TSR and DR in colorectal cancer.

### Stromal Maturity and Proportion Score (SMAPS)

To enhance the potential clinical utility and prognostic accuracy of stromal assessment, we introduced a novel Stromal Maturity and Proportion Score (SMAPS). This classification system combined TSR for the tumour centre and DR (specifically, myxoid stroma) for the invasive margin to provide a more comprehensive stromal assessment. Our findings indicated that immature DR had distinctly stronger prognostic significance than intermediate DR, comparable to stroma-high TSR (Figure 1, Table 2). Based on this, SMAPS was designed to incorporate TSR and myxoid stroma (immature DR) assessment with equal weighting. The SMAPS classification process is presented in Figure 1.

In the study cohort, 647 (59%) patients belonged to the low SMAPS group, 216 (20%) patients to the intermediate SMAPS group, and 237 (22%) patients to the high SMAPS group (Table 1). High SMAPS was significantly associated with advanced disease stage (*p* < 0.0001), high tumour grade (*p* < 0.0001), micropapillary growth pattern (*p* < 0.0001), presence of lymphovascular invasion (*p* < 0.0001), high tumour budding grade (*p* < 0.0001), SARIFA positivity (*p* < 0.0001), and MMR proficient status (*p* < 0.0001).

**Table 1.** Patient and tumour characteristics and their associations with Stromal Maturity and Proportion Score (SMAPS) in the study cohort.

		SMAPS			
Characteristic	Total *N*	Low	Intermediate	High	*p*
All cases	1100	647 (59%)	216 (20%)	237 (22%)	
Sex					
Female	543 (49%)	311 (48%)	112 (52%)	120 (51%)	0.57
Male	557 (51%)	336 (52%)	104 (48%)	117 (49%)	
Age (years)					
<65	290 (26%)	167 (26%)	45 (21%)	78 (33%)	0.007
65–75	381 (35%)	231 (36%)	68 (31%)	82 (35%)	
>75	429 (39%)	249 (38%)	103 (48%)	77 (32%)	
Year of operation					
2000–2005	342 (31%)	213 (33%)	56 (26%)	73 (31%)	0.34
2006–2010	353 (32%)	208 (32%)	71 (33%)	74 (31%)	
2011–2015	405 (37%)	226 (35%)	89 (41%)	90 (38%)	
Tumour location					
Proximal colon	536 (49%)	309 (48%)	118 (55%)	109 (46%)	0.030
Distal colon	404 (37%)	235 (36%)	66 (31%)	103 (43%)	
Rectum	160 (15%)	103 (16%)	32 (15%)	25 (11%)	
Disease stage					
I	184 (17%)	166 (26%)	16 (7%)	2 (1%)	<0.0001
II	408 (37%)	285 (44%)	71 (33%)	52 (22%)	
III	355 (32%)	148 (23%)	94 (44%)	113 (48%)	
IV	153 (14%)	48 (7%)	35 (16%)	70 (30 %)	
Tumour grade					
Low-grade	882 (80%)	557 (86%)	143 (66%)	182 (77%)	<0.0001
High-grade	218 (20%)	90 (14%)	73 (34%)	55 (23%)	
Growth pattern					
Medullary	21 (2%)	18 (3%)	3 (1%)	0 (0%)	<0.0001
Micropapillary	70 (6%)	13 (2%)	26 (12%)	31 (13%)	
Mucinous	76 (7%)	52 (8%)	9 (4%)	15 (6%)	
Signet ring	28 (3%)	8 (1%)	14 (6%)	6 (3%)	
Adenocarcinoma NOS	905 (83%)	556 (86%)	164 (76%)	185 (78%)	
Lymphovascular invasion					
No	858 (79%)	573 (89%)	155 (72%)	130 (55%)	<0.0001
Yes	242 (22%)	74 (11%)	61 (28%)	107 (45%)	
Tumour budding					
Bd1 (0–4)	827 (75%)	561 (87%)	150 (69%)	116 (49%)	<0.0001
Bd2 (5–9)	156 (14%)	61 (9%)	35 (16%)	60 (25%)	
Bd3 (≥10)	117 (11%)	25 (4%)	31 (14%)	61 (26%)	
SARIFA status					
Negative	774 (70%)	594 (92%)	127 (59%)	53 (22%)	<0.0001
Positive	326 (30%)	53 (8%)	89 (41%)	184 (78%)	
MMR status					
Proficient	931 (85%)	531 (82%)	174 (81%)	226 (95%)	<0.0001
Deficient	169 (15%)	116 (18%)	42 (19%)	11 (5%)	
*BRAF* status^A^					
Wild-type	916 (83%)	540 (84%)	169 (79%)	207 (87%)	0.044
Mutant	182 (17%)	106 (16%)	46 (21%)	30 (13%)	

Abbreviations: MMR, Mismatch repair

^A^Data missing for 2 cases

**Table 2.** Univariable and multivariable Cox regression models for cancer-specific survival and overall survival according to TSR, DR classification and SMAPS in CRC.

		Colorectal cancer-specific survival			Overall survival		
Variable	No. of cases	No. of events	Univariable HR (95% CI)	Multivariable HR (95% CI)	No. of events	Univariable HR (95% CI)	Multivariable HR (95% CI)
TSR							
Stroma-low	721	138	1 (referent)	1 (referent)	322	1 (referent)	1 (referent)
Stroma-high	342	158	3.04 (2.42–3.83)	1.49 (1.15–1.93)	209	1.80 (1.51–2.15)	1.18 (0.97–1.44)
*p*			<0.0001	0.003		<0.0001	0.097
DR classification							
Mature	599	101	1 (referent)	1 (referent)	252	1 (referent)	1 (referent)
Intermediate	149	35	1.57 (1.07–2.30)	1.11 (0.75–1.66)	73	1.35 (1.04–1.75)	1.06 (0.81–1.39)
Immature	318	160	3.97 (3.09–5.10)	1.84 (1.39–2.45)	112	2.16 (1.80–2.60)	1.40 (1.13–1.74)
*P_trend_*			<0.0001	<0.0001		<0.0001	0.002
SMAPS							
Low	630	101	1 (referent)	1 (referent)	265	1 (referent)	1 (referent)
Intermediate	206	72	2.63 (1.94–3.55)	1.50 (1.08–2.07)	117	1.70 (1.36–2.11)	1.17 (0.92–1.47)
High	227	123	4.64 (3.57–6.05)	2.01 (1.47–2.75)	149	2.27 (1.85–2.77)	1.42 (1.12–1.81)
*P_trend_*			<0.0001	<0.0001		<0.0001	0.004

Abbreviations: CI, confidence interval; HR, hazard ratio

Multivariable Cox proportion hazards regression models were adjusted for sex, age (<65, 65–75, >75), year of operation (2000–2005, 2006–2010, 2011–2015), tumour location (proximal colon, distal colon, rectum), disease stage (I-II, III, IV), tumour grade (low-grade, high-grade), lymphovascular invasion (negative, positive), tumour budding (Bd1, Bd2, Bd3), mismatch repair (MMR) status (proficient, deficient) and *BRAF* status (wild-type, mutant).

*P_trend_* values were calculated by using the three categories of stromal features as continuous variables in univariable and multivariable Cox proportional hazard regression models.

In the survival analyses, SMAPS was an independent prognostic parameter for cancer-specific and overall survival (Figure 1, Table 2). In the multivariable Cox regression model for cancer-specific mortality, the HR for high (vs. low) SMAPS category was 2.01 (95% CI: 1.47–2.75, *P*_trend_<0.0001), exceeding the individual HR point estimates for immature DR and stroma-high TSR. Moreover, the intermediate SMAPS category demonstrated worse prognosis compared to the intermediate DR group, suggesting that SMAPS enhances stratification compared to DR classification alone (Figure 1, Table 2, Table S3). When analyzed by stage, the prognostic effect of SMAPS was strongest in stage III patients (*p* < 0.0001) followed by stage II (*p* = 0.002) patients (Figure S4). The prognostic power of SMAPS showed a trend towards statistical significance in stage I patients (*p* = 0.025). SMAPS was not statistically significant prognostic factor in stage IV patients (*p* = 0.34).

In ROC analysis for cancer-specific survival, SMAPS showed the highest AUC (0.69, 95% CI 0.66–0.73) followed by DR classification, SARIFA, TSR, and tumour budding (Figure S5), indicating that SMAPS provided the strongest prognostic discrimination among the evaluated markers. Direct comparisons of the prognostic power of SMAPS with tumour budding and SARIFA are presented in Table S4 and Table S5. SMAPS remained as an independent prognostic factor and showed comparable or higher prognostic power than tumour budding and SARIFA in multivariable analyses. SMAPS showed performance comparable to tumour budding and was an independent prognostic factor for CSS in a subgroup analysis containing 396 stage II CRC patients (Table S6).

### Stromal features and the composition of tumour microenvironment

**Figure 3.**
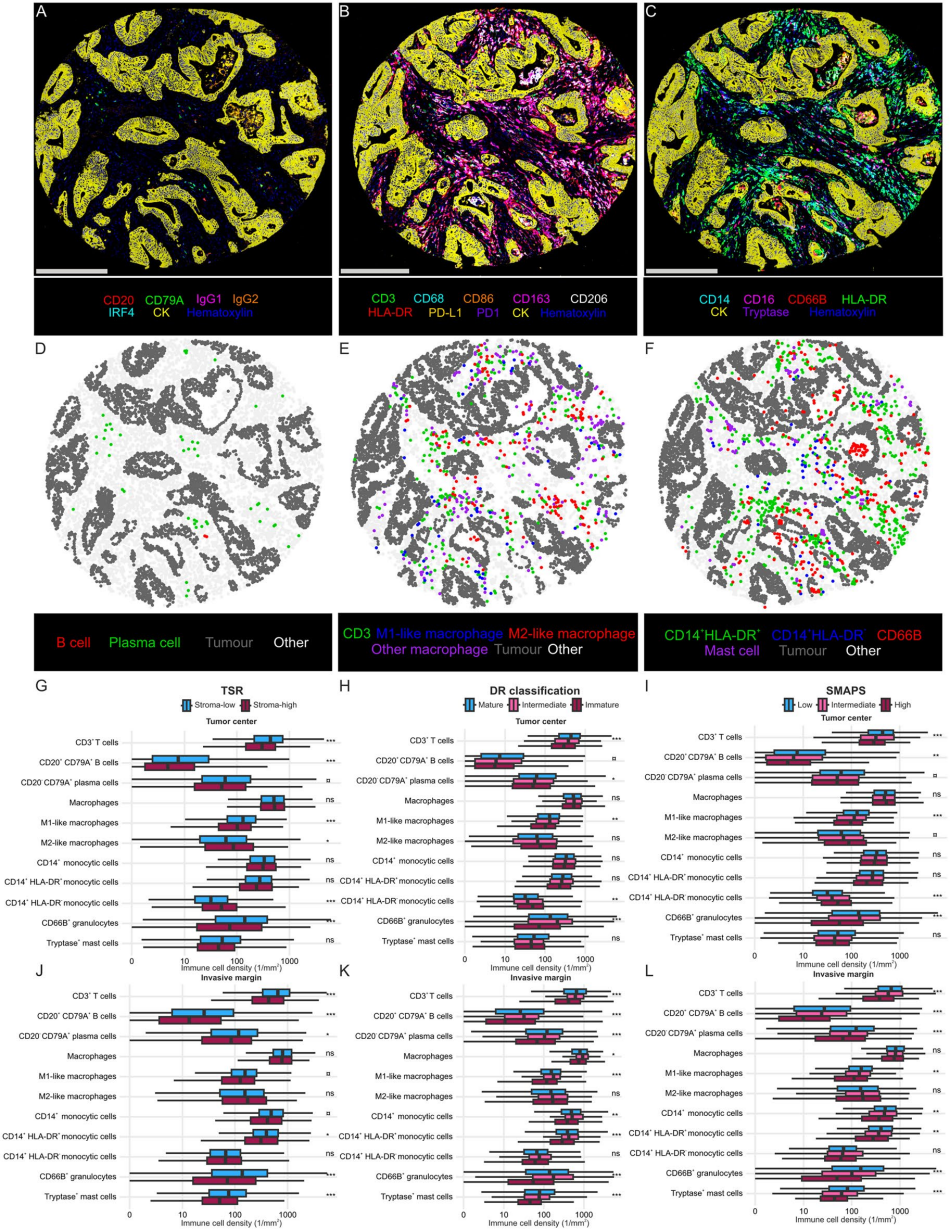
Immune cell analyses using multiplex immunohistochemistry and digital image analyses. Example images of multiplex immunohistochemistry detecting for B cells (A), macrophages (B) and myeloid cells (C). Scale bars are 300 µm. Corresponding cell maps (D-F) based on digital image analyses. Tumour centre (G-I) and invasive margin (J-L) immune cell densities are presented separately by boxplots. Immune cell densities were studied in relation to TSR (G, J), DR classification (H, K), and SMAPS (I, L). Immune cell analyses were based on cohort 1 data, in which *N* = 1043 for CD3^+^ T cells, macrophages, M1-like macrophages, and M2-like macrophages, *N* = 1052 for CD20^+^CD79A^+^ B cells and CD20^-^CD79A^+^ plasma cells, *N* = 1001 for CD14^+^ monocytic cells, CD14^+^HLA-DR^+^ mature monocytic cells, CD14^+^HLA-DR^-^ immature monocytic cells, CD66b^+^ granulocytes, and tryptase^+^ mast cells for tumour centre (G-I). In the invasive margin, *N* = 960 for CD3^+^ T cells, CD20^+^CD79A^+^ B cells, CD20^-^CD79A^+^ plasma cells, macrophages, M1-like macrophages, and M2-like macrophages, *N* = 904 for CD14^+^ monocytic cells, CD14^+^HLA-DR^+^ mature monocytic cells, CD14^+^HLA-DR^-^ immature monocytic cells, CD66b^+^ granulocytes, and tryptase^+^ mast cells. ns: *p* > 0.05, ¤: *p* = 0.05–0.005, *p < 0.005, **p < 0.001, ***p < 0.0001. Abbreviations: TSR: tumour-stroma ratio; DR: desmoplastic reaction; SMAPS: Stromal Maturity and Proportion Score.

We hypothesized that the tumours showing high TSR, immature DR, and high SMAPS might exhibit an immunosuppressive tumour microenvironment. To investigate this, immune cell densities were assessed using three multiplex immunohistochemistry assays and analyzed in relation to TSR, DR classification, and SMAPS (Figure 3). In the tumour centre, tumours in the stroma-high TSR group, the immature DR group, and the high SMAPS group were associated with lower densities of CD3^+^ T cells (*p* < 0.0001 for all), M1-like macrophages (*p* < 0.0002 for all), and CD66B^+^ granulocytes (*p* < 0.0001 for all). The associations in the invasive margin were statistically significant for lower densities of CD3^+^ T cells (*p* < 0.0001 for all), CD20^+^CD79A^+^ B cells (*p* < 0.0001 for all), CD20^-^CD79A^+^ plasma cells (*p* < 0.002 for all), M1-like macrophages (<0.005 for all), CD66B^+^ granulocytes (*p* < 0.0001 for all), and tryptase^+^ mast cells (*p* < 0.0001 for all). Additionally, in the tumour centre, the stroma-high TSR displayed higher densities of M2-like macrophages (*p* < 0.005).

Monocytic cell composition also varied according to TSR, DR, and SMAPS. In the tumour centre, CD14^+^HLA-DR^-^ immature monocytic cells (*p* < 0.0002 for all) were more prevalent in tumours with high TSR, immature DR, and high SMAPS. Conversely, in the invasive margin, CD14^+^HLA-DR^+^ mature monocytic cells (*p* < 0.004 for all) were present at lower densities in the same tumour groups.

Given the strong correlation between SMAPS and immune cell infiltration, we evaluated its prognostic significance in comparison to the Immune cell score using Cox regression models Table S7. High SMAPS remained an independent predictor of higher CRC mortality in multivariable models that included the Immune cell score.

### Digital image analysis of myxoid stroma using Alcian blue staining

**Figure 4.**
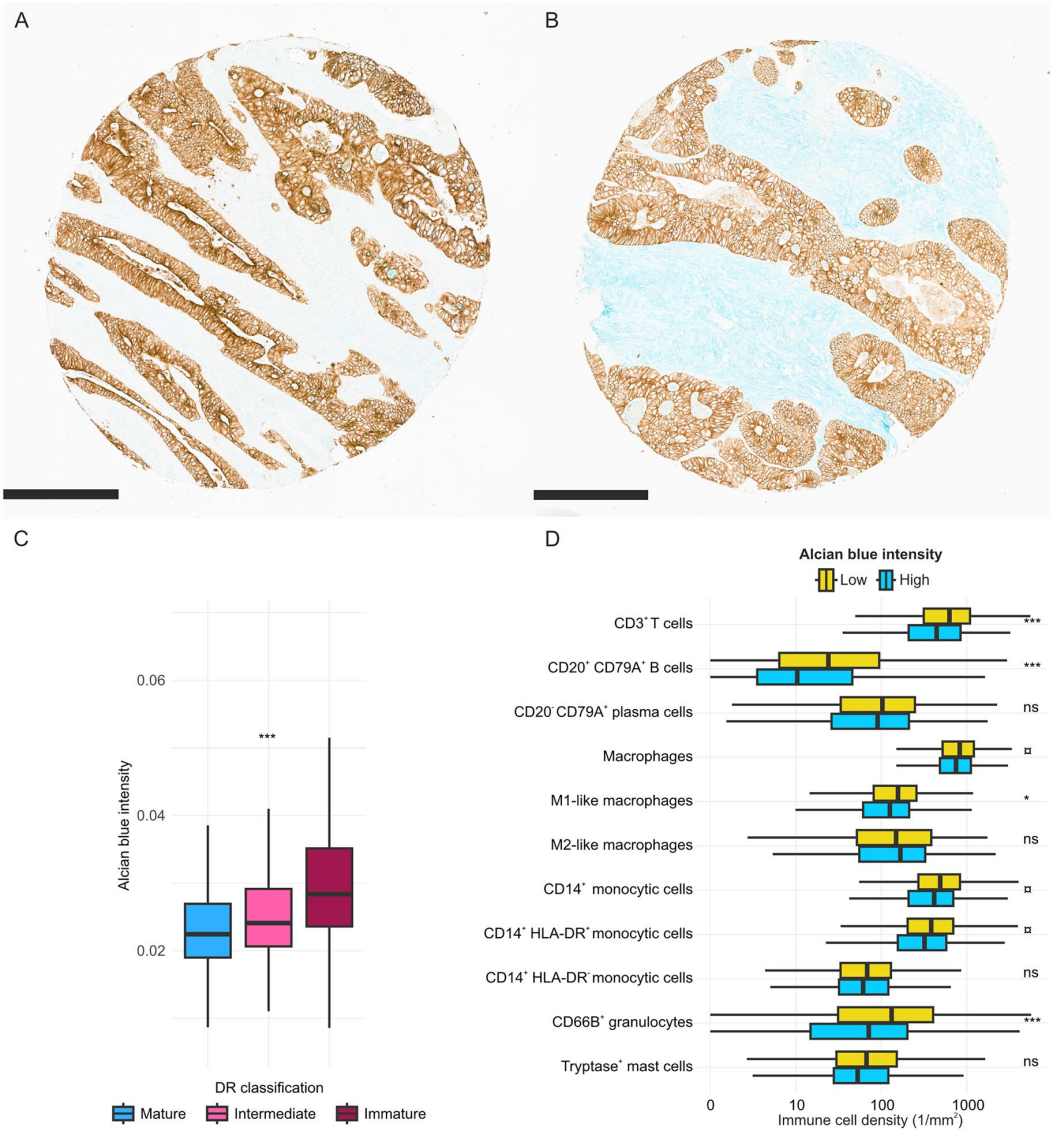
Alcian blue staining in relation to desmoplastic reaction classification and immune cell densities. Example images of TMA cores with low (A) and high (B) Alcian blue intensities. Scale bars are 300 µm. Associations between Alcian blue intensities and desmoplastic reaction (DR) classifications are shown in boxplots (C). Immune cell densities at the invasive margin categorized by stromal Alcian blue intensity are displayed as boxplots (D). Cut-off value for high alcian blue intensity was 0.03 intensity units. Immune cell analyses were based on cohort 1 data including alcian blue intensity, with *N* = 939 for CD3^+^ T cells, macrophages, M1-like macrophages, and M2-like macrophages, *N* = 940 for CD20^+^CD79A^+^ B cells and CD20^-^CD79A^+^ plasma cells, *N* = 884 for CD14^+^ monocytic cells, CD14^+^HLA-DR^+^ mature monocytic cells, CD14^+^HLA-DR^-^ immature monocytic cells, CD66B^+^ granulocytes, and tryptase^+^ mast cells. ns: *p* > 0.05, ¤: *p* = 0.05–0.005, *p < 0.005, **p < 0.001, ***p < 0.0001.

To more objectively assess myxoid stroma, we employed digital image analysis to measure the intensity of Alcian blue staining (Figure 4). Tumours classified within the immature DR group, characterized by prominent myxoid stroma, exhibited significantly higher Alcian blue intensities (*p* < 0.0001). High Alcian blue intensity in the invasive margin was associated with lower densities of several immune cell populations in the invasive margin, including CD3+ T cells (*p* < 0.0001), CD20^+^CD79A^+^ B cells (*p* < 0.0001), M1-like macrophages (*p* = 0.0028), CD14^+^HLA-DR^+^ mature monocytic cells (*p* = 0.0045) and CD66b^+^ granulocytes (*p* < 0.0001), aligning with the results observed for the myxoid stroma evaluation from the H&E-stained slides.

### Validation cohort

To validate the prognostic significance of SMAPS and its relationship with T cell infiltration, a separate cohort of 776 patients was analyzed. Among these patients, 362 (47%) belonged to the low SMAPS group, 284 (37%) to the intermediate SMAPS group, and 130 (17%) to the high SMAPS group (Table S8). High SMAPS was associated with advanced disease stage (*p* < 0.0001), micropapillary growth pattern (<0.0001), presence of lymphovascular invasion (*p* < 0.0001), higher tumour budding grade (*p* < 0.0001), SARIFA positivity (*p* < 0.0001), and MMR proficient status (<0.0001).

**Figure 5.**
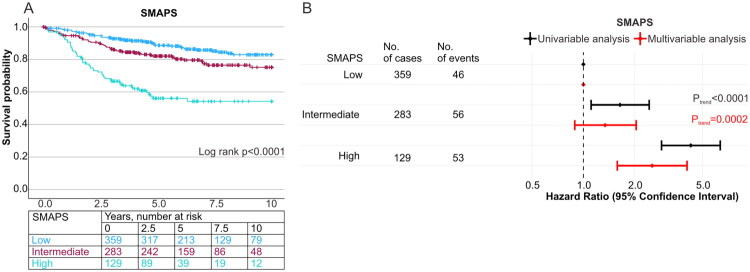
Stromal Maturity and Proportion Score (SMAPS) and survival in the validation cohort. Kaplan-Meier curves for cancer-specific survival by SMAPS are displayed (A). Numbers of cases and events of SMAPS in validation cohort are shown in table (B). Cox regression models including univariable and multivariable analyses of SMAPS are presented as a forest plot (B). Multivariable Cox proportion hazards regression models were adjusted for sex, age (<65, 65–75, >75), year of operation (2006–2010, 2011–2015, 2016–2020), tumour location (proximal colon, distal colon, rectum), disease stage (I-II, III, IV), tumour grade (low-grade, high-grade), lymphovascular invasion (negative, positive), tumour budding (Bd1, Bd2, Bd3), mismatch repair (MMR) status (proficient, deficient), and *BRAF* status (wild-type, mutant).

Kaplan-Meier estimates demonstrated that high SMAPS was associated with shorter 10-year cancer-specific survival (*p* < 0.0001) (Figure 5). Multivariable Cox regression confirmed that high SMAPS was associated with higher cancer-specific mortality (Figure 5, Table S9). The multivariable HR for high (vs. low) SMAPS was 2.53 (95% CI: 1.58–4.05). The results of stage II subgroup analyses for the validation cohort are presented in Table S6. The associations between adjuvant therapy status (no vs. yes) and survival were similar in patients with low vs. intermediate/high SMAPS in stage II (P_interaction_=0.96) and stage III patients (P_interaction_=0.98) (Figure S6). High SMAPS was also associated with lower T cell densities in both the tumour centre and invasive margin, consistent with findings from the study cohort (Table S10).

## Discussion

In this study of two CRC cohorts (*N* = 1,876), we introduced SMAPS, a stromal scoring system incorporating TSR and DR classification that outperformed individual variables in predicting cancer-specific survival. High SMAPS, stroma-high TSR, and immature DR were linked to an immunosuppressive tumour microenvironment with lower antitumour immune cell densities. High Alcian blue staining intensity was further associated with the same immunosuppressive microenvironmental features observed in tumours with myxoid stroma. These findings highlight the prognostic value of stromal assessment and its potential to refine risk stratification in CRC.

We found that both TSR and DR were significantly associated with shorter cancer-specific survival in stage I-IV CRC, in line with previous reports of TSR [4,13,15,36,37] and immature DR [6,9,22,38] as adverse prognostic features. To refine stromal evaluation, we introduced SMAPS, combining TSR in the tumour centre and myxoid stroma at the invasive margin. SMAPS outperformed TSR and DR classification, as single variables, identifying a smaller high-risk group with particularly poor outcomes and a larger intermediate-risk group with worse prognosis than the intermediate DR group. High SMAPS was also a stronger predictor of adverse outcome than tumour budding in stage I-IV patients, overall, while in stage II disease, the HR point estimates of SMAPS and tumour budding were comparable, consistent with prior data suggesting that DR-based biomarkers can be at least as informative as tumour budding in this setting [21,24,39].

When analyzed by stage, the prognostic effect of SMAPS was strongest in stage III, followed by stage II, associated towards statistical significance in stage I, and was not significant in stage IV disease. This pattern suggests that stromal architecture in the primary tumour is most informative for risk stratification in non-metastatic CRC. In stage II-III patients, where decisions regarding adjuvant chemotherapy may be difficult, SMAPS may help refine risk estimation and support discussions about treatment intensity and follow-up. In the validation cohort, the association between adjuvant treatment status and survival was similar in patients with low vs. intermediate/high SMAPS in both stage II and III disease, supporting a primarily prognostic rather than predictive role for SMAPS. Prospective and treatment-stratified studies are needed to evaluate how SMAPS might be integrated into routine decision-making.

Multiplex immunohistochemistry combined with digital image analysis provided a high-resolution assessment of immune cell densities in relation to TSR, DR, and SMAPS classifications. This approach enabled the analysis of detailed cellular phenotypes and revealed an immunosuppressive microenvironment associated with stroma-high TSR, immature DR, and high SMAPS. These groups exhibited lower densities of key antitumour immune cells, including T cells, M1-like macrophages, and CD66B^+^ granulocytes. Our findings align with previous studies linking stroma-high TSR to reduced immune cell infiltration [37] and linking immature stroma to lower densities of intraepithelial memory cytotoxic T cells and stromal M1-like macrophages in CRC [22]. In addition to the lower densities of M1-like macrophages, stroma-high TSR in the tumour centre was associated with higher densities of M2-like macrophages. While classically activated M1-macrophages are proinflammatory, alternatively activated M2-like macrophages are anti-inflammatory and often promote tumour progression [40]. Higher densities of M2-like macrophages have been linked to poor prognosis in CRC [41,42], consistent with our observation that stroma-high TSR was associated with worse prognosis compared with stroma-low TSR.

Our study also demonstrated a strong association between stromal features and monocytic cell maturity, as stroma-high TSR, immature DR, and high SMAPS were associated with higher densities of CD14^+^HLA-DR^-^ immature monocytic cells in tumour centre but lower densities of the CD14^+^HLA-DR^+^ mature monocytic cells in the invasive margin. The accumulation of immature monocytic cells in the tumour centre may reflect impaired myeloid cell maturation driven by hypoxia and elevated immunosuppressive cytokine levels [43]. Notably, these immature monocytic cells may include myeloid-derived suppressor cells, which contribute to immune evasion, angiogenesis, and tumour progression [44]. These findings highlight the role of stromal remodelling in shaping immune cell infiltration and suggest that stromal features could serve as indirect markers of an immunosuppressive tumour milieu.

High TSR, immature DR and high SMAPS were associated with other strong predictors of adverse outcomes, including tumour budding and the recently described SARIFA [28,45]. Despite this overlap, SMAPS remained as an independent prognostic marker in multivariable models, suggesting it captures complementary aspects of the stromal-tumour interactions. We did not assess the relationships between SMAPS and the Glasgow Microenvironment Score or the Klintrup-Mäkinen (K-M) Score. However, a low K-M score has been associated with similar characteristics as high SMAPS category, including lower T cell densities and advanced stage [46], suggesting that high SMAPS may correspond to a more suppressed immune microenvironment at the invasive front. The Glasgow Microenvironment Score integrates TSR and the K-M score into a three-tier system (GMS 0–2), where GMS 2 (high TSR and low K-M score) is associated with worse prognosis in stage I-III CRC [47]. High TSR, immature DR and high SMAPS were all linked to micropapillary growth pattern, which is also associated with worse prognosis [48]. Aligning with our immune cell findings in high TSR, immature DR, and high SMAPS groups, micropapillary growth pattern has also been associated with lower densities of CD3^+^ T cells and CD14^+^HLA-DR^+^ mature monocytic cells [48].

The mechanisms underlying these invasion front biomarkers are not yet fully understood, but several lines of evidence suggest partially shared biological pathways. Myxoid stroma and keloid-like collagen, key features of immature DR, are produced by CAFs [10]. Stroma-high TSR has been linked to increased expression of CAF markers in CRC [49]. Tumour cells can instruct CAFs through paracrine signalling, and CAFs can promote epithelial-mesenchymal transition (EMT) by secreting factors such as tenascin-C [50], while tumour budding is strongly associated with EMT [51]. Together, these data suggest that TSR, DR classification, and tumour budding may be interconnected through tumour cell-driven, CAF-mediated EMT and stromal remodelling. The prognostic power of SARIFA has been attributed to tumour-adipocyte interactions and associated immune alterations in SARIFA-positive tumours [52,53]. SMAPS, by integrating TSR and myxoid stroma at the invasive margin, may therefore reflect combined effects of CAF activation, EMT, and immune exclusion. Further mechanistic studies are needed to investigate these overlapping pathways in more detail.

We utilized Alcian blue stain, which binds to acidic polysaccharides, and digital image analysis to more quantitatively evaluate myxoid stroma. As expected, high Alcian blue intensity was associated with the immature DR group characterized by myxoid stroma [10]. This is likely explained by the high glycosaminoglycan content of myxoid stroma, which is readily detected by Alcian blue staining [54]. However, the intermediate DR group which lacks overt myxoid stroma on H&E, showed slightly higher Alcian blue intensities than the mature DR group. This pattern may partly reflect the broad distribution of Alcian blue intensities within DR categories defined on H&E whole-slide sections and differences between whole-slide and TMA sampling. A more pronounced distinction between the DR groups might have been achieved by specifically selecting TMA cores from the most myxoid regions. Notably, using multiplex immunohistochemistry, we found that high Alcian blue intensity was associated with an immunosuppressive microenvironment, mirroring the immune cell associations observed for immature stroma evaluated from H&E-stained sections. However, overall, there are currently insufficient data on the added value of Alcian blue staining over H&E assessment to support its routine use in determining myxoid stroma in CRC. Nevertheless, the potential value of Alcian blue staining should be further evaluated in whole-slide sections and, particularly in stroma-rich tumour types, such as pancreatic ductal adenocarcinoma.

Stromal parameters can be visually assessed on routine H&E-stained sections without special equipment, which facilitates implementation in everyday practice worldwide. However, interobserver variability remains an important limitation. In line with our findings, previous studies have reported substantial to almost perfect agreement for TSR [55,56] and fair to substantial agreement for DR classification [57,58]. Deep learning algorithms have been studied to quantify TSR and DR in whole slide images, showing strong correlation with microscopic assessment (for TSR) [59] and robust prognostic value [60–62]. In our study, SMAPS showed substantial reproducibility (Cohen’s kappa 0.67), supporting its potential for routine visual use. At the same time, the existing evidence for deep learning based TSR and DR assessment suggests that SMAPS could also be captured by deep learning models, either as a fully automated score or in a hybrid workflow where deep learning pre-segmentation and quantification support and standardize pathologist scoring.

Our study has some limitations to consider. Immune cell densities were examined from tissue microarrays, which do not completely represent the heterogeneity of the whole tumour. Similarly, Alcian blue intensity was analyzed from the same tissue microarray cores, meaning the most myxoid areas might not have been captured, potentially underestimating the extent of myxoid stroma. Multiple immune cells were analyzed in the study, which could increase the risk of type I error. To mitigate this, we applied a stringent significance threshold of *p* < 0.005, as recommended by an expert panel [35]. Nevertheless, some risk of false-positive findings remains, and the results of the immune cell analyses, particularly those close to this threshold, should be interpreted cautiously and validated in independent cohorts. TSR, DR, and tumour budding were evaluated from a single slide section with the deepest invasion in the study cohort, whereas multiple slides per case were assessed in the validation cohort. Interobserver agreement for single slide evaluations compared to multiple slides ranged from substantial to almost perfect. A previous study also showed excellent agreement between single-slide and multi-slide evaluations for DR and tumour budding [39], though TSR was not included. Additionally, patients who had received preoperative treatments were excluded and treatment for recurrence data was not available. Therefore, further studies are needed to determine whether these findings apply to neoadjuvant treated cases and to examine the impact of treatment for recurrence on prognosis. Data on adjuvant treatment were only available for the validation cohort. Although we did not find evidence that the effect of adjuvant treatment differed by SMAPS status in stage II or III patients, these analyses are based on a single cohort and should be validated in independent datasets. Transcriptomic studies of SMAPS were not included in this study. Future studies integrating SMAPS with RNA-based profiling could help characterize the underlying molecular programs associated with different SMAPS categories and to better elucidate the biology of the stromal microenvironment.

## Conclusions

TSR and DR independently predicted cancer-specific survival, while SMAPS emerged as a stronger prognostic indicator by integrating TSR and myxoid stroma assessment. Stroma-high TSR, immature DR, and high SMAPS were linked to an immunosuppressive microenvironment, highlighting the impact of stromal alterations on tumour-immune interactions. These findings support SMAPS as a valuable tool for improving risk assessment in CRC.

## Supplementary Material

Supplemental Material

Supplementary figures.zip

supplementary tables.zip

## Data Availability

The data generated and/or analyzed during this study are not publicly available due to confidentiality of patient data. The sharing of data will require approval from relevant ethics committees and/or biobanks. Further information including the procedures to obtain and access data of Finnish Biobanks are described at https://finbb.fi/en/fingenious-service. Additional questions can be directed to the corresponding author (JPV).
