# Supplementary material for: Impact of stromal maturity and proportion on prognosis and immune landscape in colorectal cancer
Source: Ann Med. 2025 Dec 26;58(1):2606512. doi: 10.1080/07853890.2025.2606512 (PMC12777758; doi:10.1080/07853890.2025.2606512)
Supplement: Supplemental Material [file IANN_A_2606512_SM4298.docx]

Figure S1. Flow of patients through the study.

Figure S2 Example images (A-C) of discrepant cases in SMAPS evaluation. In case A, the discrepancy was between SMAPS low and intermediate categories due to difference in TSR evaluation. The evaluators disagreed on whether the most stroma-rich spot (D) filled the published criteria of having vital tumor cells on all four sides of the field of vision. Evaluators agreed on the lack of myxoid stroma (E). In case B, the discrepancy was between SMAPS low and intermediate categories. Both evaluators assessed the TSR (F) as stroma-low. Evaluators differed on opinion whether the stroma (G) was myxoid or not, highlighting the subjectivity of definition of myxoid. In case C, the discrepancy was between SMAPS intermediate and high categories. Evaluators disagreed on whether the spot (H) was stroma-high or stroma-low. Evaluators agreed on the presence of myxoid stroma (I). The scale bars are 5.0 mm. The diameter of pictures (D, F, H) is 2.0 mm.

Figure S3. Multiplex immunohistochemistry protocol and primary antibodies. Abbreviations: IHC, immunohistochemistry; AEC, 3-Amino-9-ethylcarbazole 1Dewax solution (AR9222, Leica Biosystems), 30min, 60–72 ˚C. 2Epitope retrieval done with BOND epitope retrieval solution 2 (EDTA based, pH 9, AR9640, Leica Biosystems) with 20min heating time. 3Primary antibodies (name, clone, catalogue number and manufacturer) together with the selected antigen retrieval conditions and used dilutions are listed in the staining order. 4AEC + high sensitivity substrate (K3469, Dako). 5VectaMount AQ Aqueous Mounting Medium (H-5501, Vector Laboratories), digitized with a 20x objective using NanoZoomer XR (Hamamatsu) slide scanner. 6The sections were destained with ethanol and antibody stripping was done with heat-induced epitope retrieval using BOND epitope retrieval solution 1 (citrate based, pH 6, AR9961, Leica Biosystems) or 2. Suitable epitope retrieval conditions were optimized for each antibody.

Figure S4. Kaplan-Meier curves for cancer-specific survival according to SMAPS within each stage.

Figure S5. Receiver operating characteristics (ROC) analysis for cancer-specific survival, comparing the discriminative performance of histological prognostic markers. The analysis included tumor budding (AUC=0.618), stromal maturity and proportion score (SMAPS) (AUC=0.694), SARIFA (AUC=0.662), tumor-stroma ratio (TSR) (AUC=0.647), and desmoplastic reaction (DR) classification (AUC=0.681).

Figure S6. Interaction between SMAPS and adjuvant therapies in the validation cohort. Kaplan-Meier survival curves according to adjuvant therapy (no vs. yes) are presented in SMAPS low (A, C) and SMAPS intermediate & high (B, D) categories. Stage 2 patients are shown in panels A-B and stage 3 patients are shown in panels C-D. Altogether N=221 received adjuvant treatment (49 in stage II and 172 in stage III). 120 patients receiving oxaliplatin based therapy (e.g., XELOX or FOLFOX), 86 patients receiving fluoropyrimidine alone, and 15 patients receiving other treatments (e.g., chemoradiotherapy). Patients who died in less than 30 days after surgery were excluded from the analyses. Pinteraction values were calculated using the Wald test for the cross product of SMAPS (low vs. intermediate/high) and adjuvant treatment status (no vs. yes) in Cox regression models

Table S1. Patient and tumor characteristics and their associations with tumor-stroma ratio (TSR)

Table S2. Patient and tumor characteristics and their associations with Desmoplastic reaction (DR) classification

Table S3. Multivariable Cox regression models for cancer-specific survival

Table S4. Comparison of the prognostic power of SMAPS and tumor budding using Cox regression models for cancer-specific survival.

Table S5. Comparison of the prognostic power of SMAPS and SARIFA using Cox regression models for cancer specific survival.

Table S6. The prognostic power of tumor-stroma ratio (TSR), desmoplastic reaction (DR) classification, the Stroma Maturity and Proportion Score (SMAPS), and tumor budding in stage II colorectal cancer patients using Cox regression models for cancer-specific survival.

Table S7. Comparison of the prognostic power of SMAPS and Immune cell score using Cox regression models for cancer-specific survival.

Table S8. Patient and tumor characteristics and their associations with Stroma Maturity and Proportion Score (SMAPS) in the validation cohort

Table S9. Multivariable Cox regression models for cancer-specific survival in the validation cohort.

Table S10. T cells densities according to the Stroma Maturity and Proportion Score (SMAPS) in the validation cohort.
