# Supplementary figures and images for: Impact of stromal maturity and proportion on prognosis and immune landscape in colorectal cancer
Source: Ann Med. 2025 Dec 26;58(1):2606512. doi: 10.1080/07853890.2025.2606512 (PMC12777758; doi:10.1080/07853890.2025.2606512)

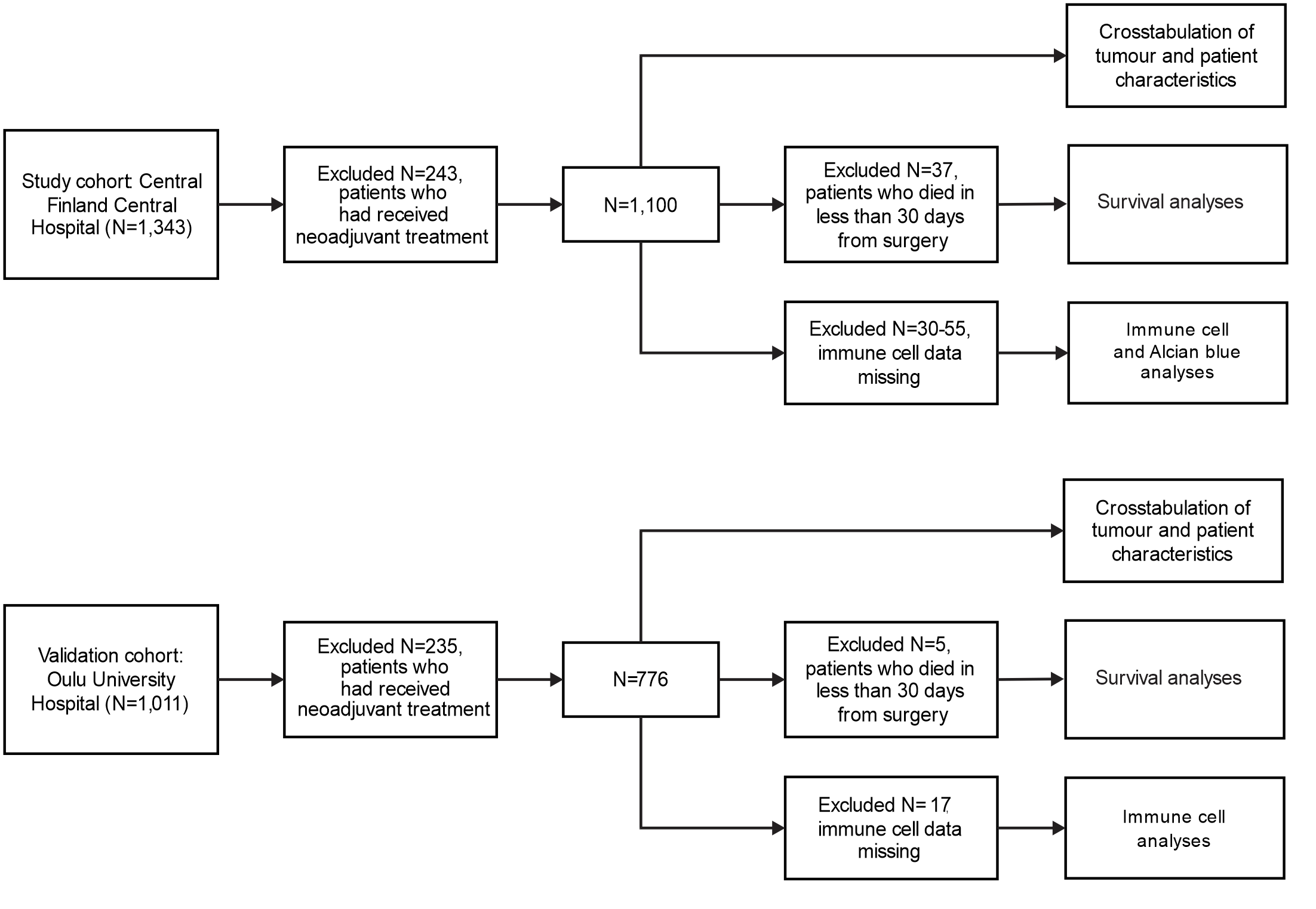

Supplement: Supplementary figures.zip [file IANN_A_2606512_SM3391.zip › FigureS1.tif]

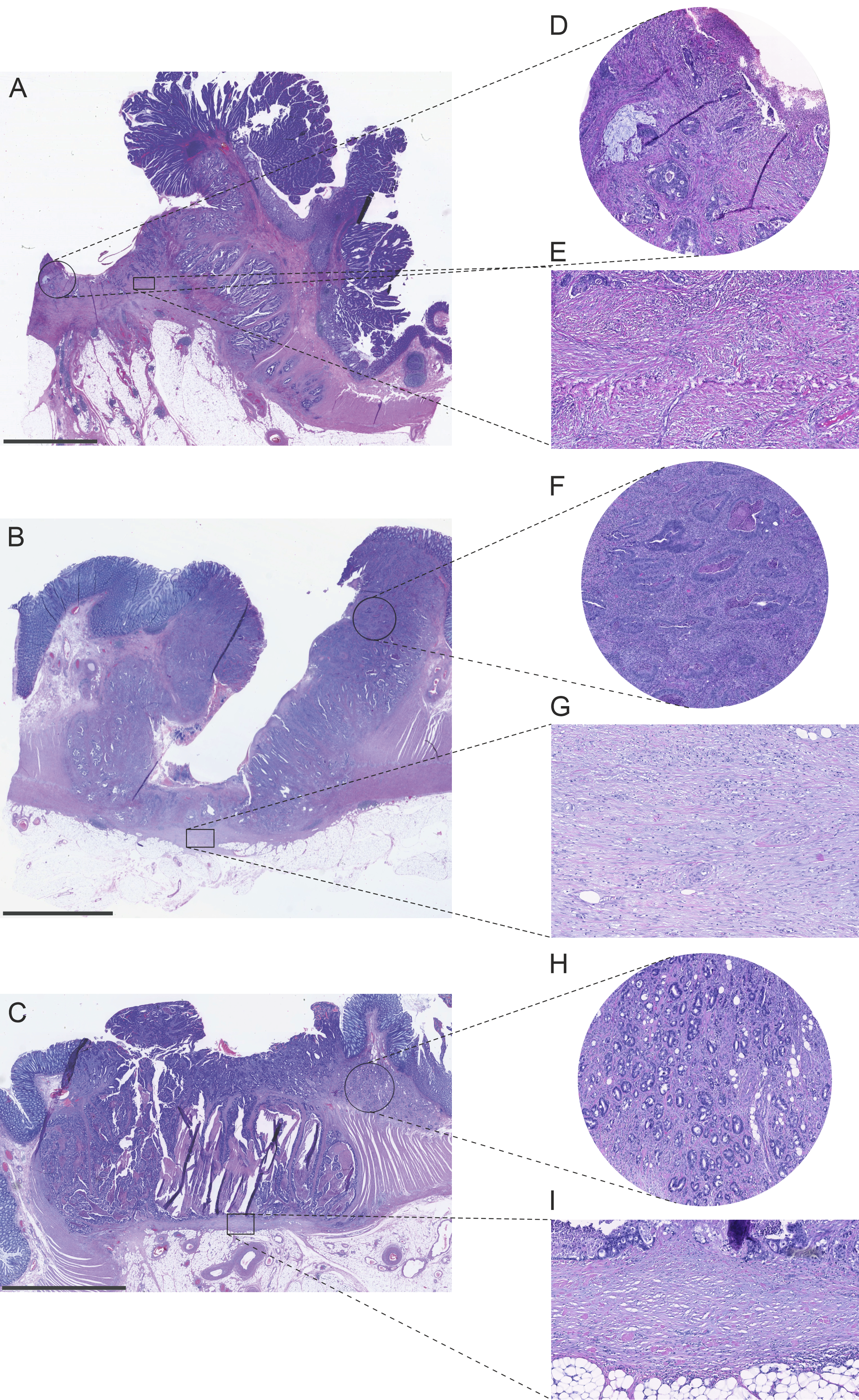

Supplement: Supplementary figures.zip [file IANN_A_2606512_SM3391.zip › FigureS2.tif]

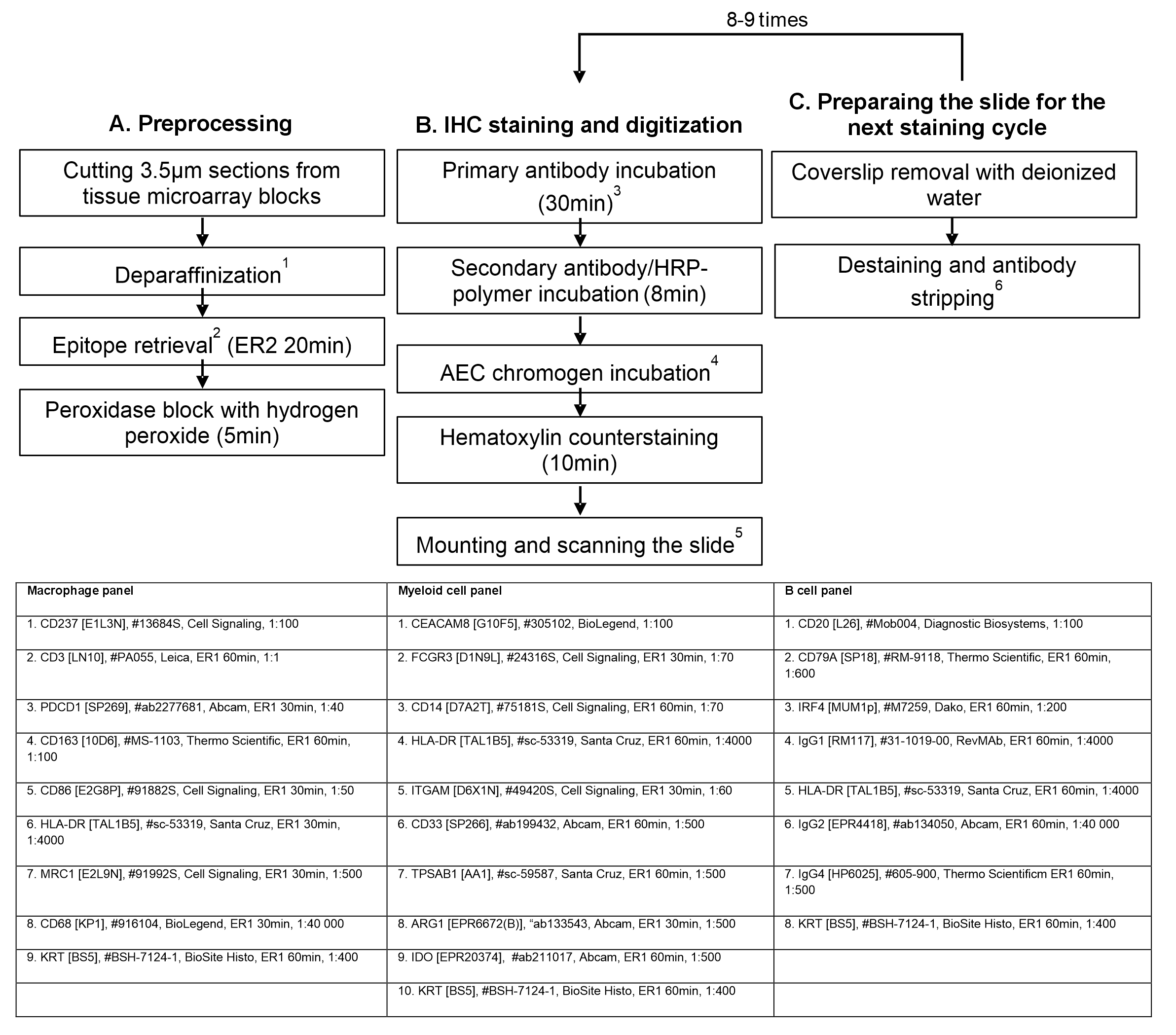

Supplement: Supplementary figures.zip [file IANN_A_2606512_SM3391.zip › FigureS3.tif]

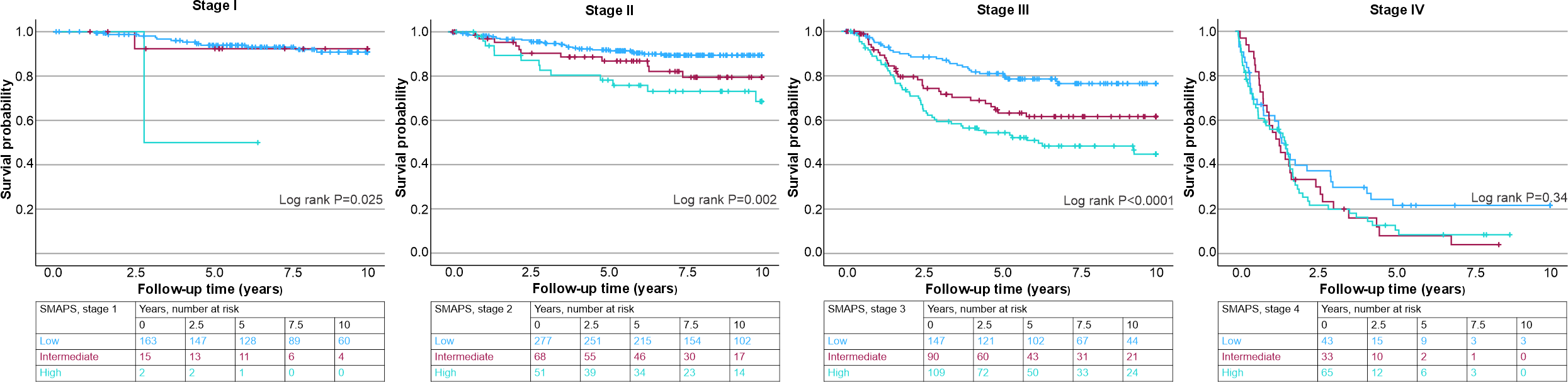

Supplement: Supplementary figures.zip [file IANN_A_2606512_SM3391.zip › FigureS4.tif]

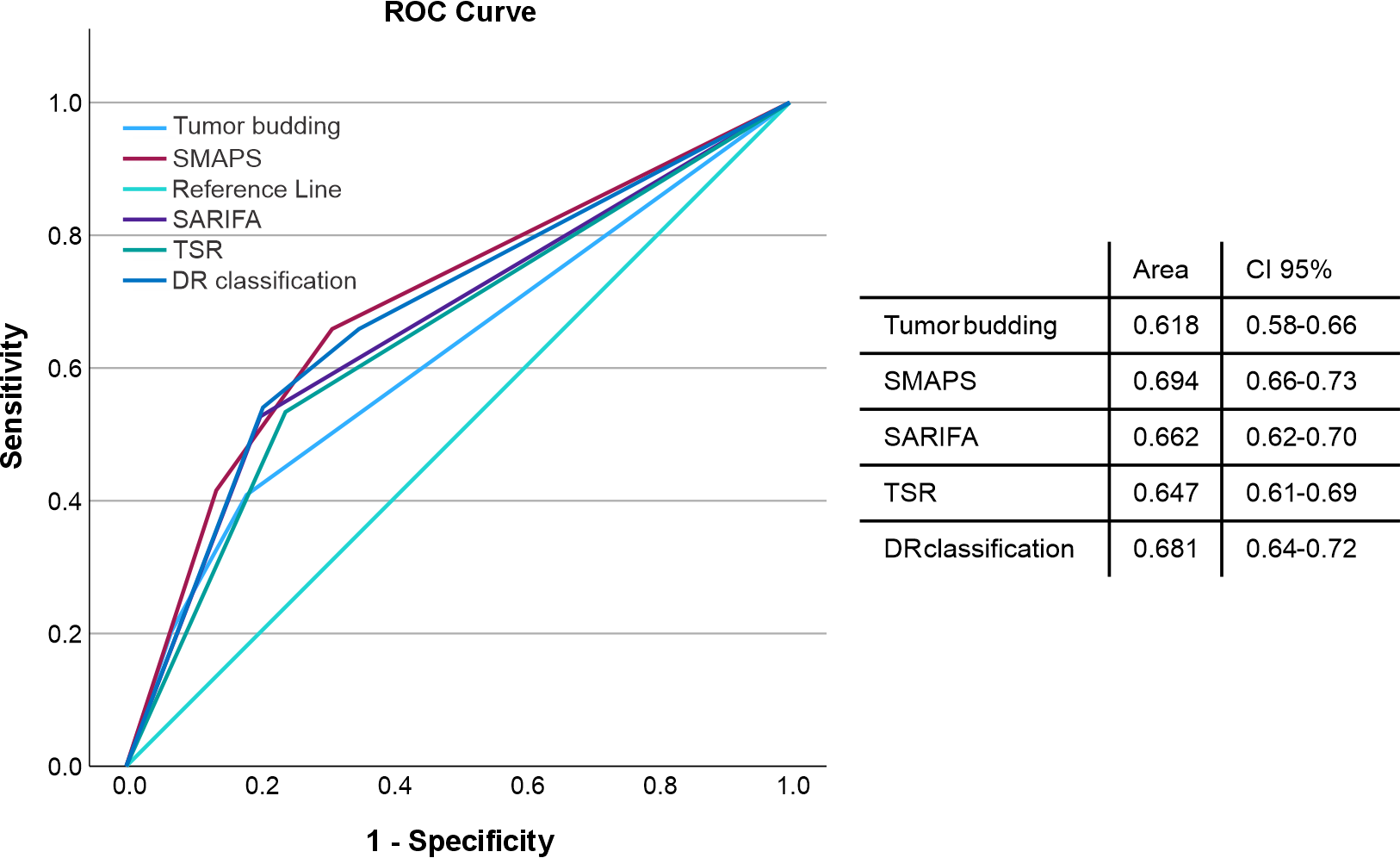

Supplement: Supplementary figures.zip [file IANN_A_2606512_SM3391.zip › FigureS5.tif]

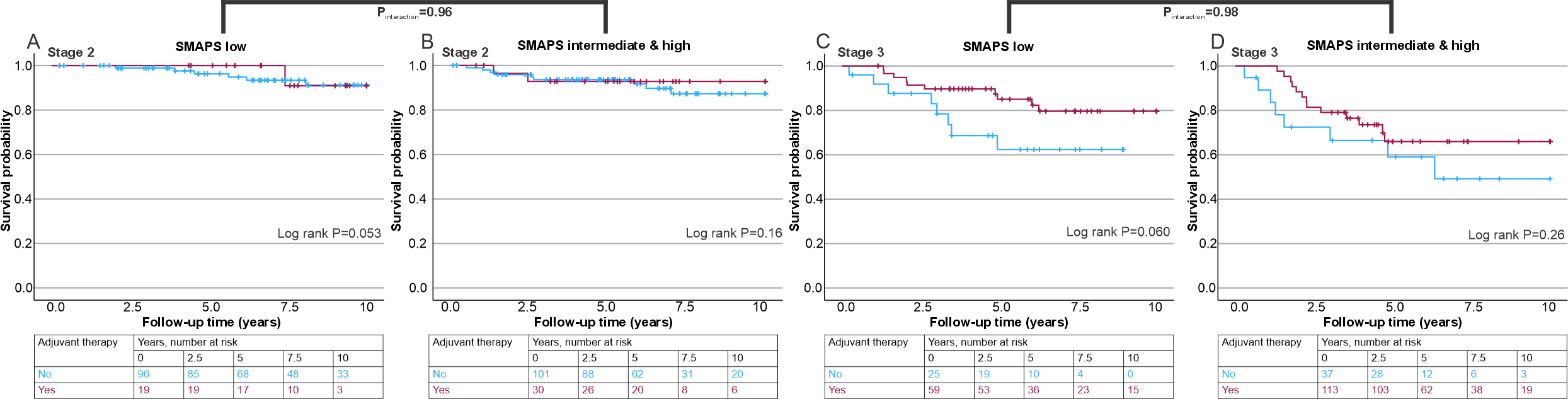

Supplement: Supplementary figures.zip [file IANN_A_2606512_SM3391.zip › FigureS6.tif]
