# Supplementary material for: Impact of stromal maturity and proportion on prognosis and immune landscape in colorectal cancer
Source: Ann Med. 2025 Dec 26;58(1):2606512. doi: 10.1080/07853890.2025.2606512 (PMC12777758; doi:10.1080/07853890.2025.2606512)
Supplement: supplementary tables.zip [file IANN_A_2606512_SM3390.zip › TableS1.docx]

|  |  | TSR | |  |
| --- | --- | --- | --- | --- |
| Characteristic | Total N | Stroma-low | Stroma-high | P |
| All cases | 1100 | 743 (68%) | 357 (32%) |  |
| Sex |  |  |  |  |
| Female | 543 (49%) | 362 (49%) | 181 (51%) | 0.54 |
| Male | 557 (51%) | 381 (51%) | 176 (49%) |  |
| Age (years) |  |  |  |  |
| <65 | 290 (26%) | 185 (25%) | 105 (29%) | 0.20 |
| 65-75 | 381 (35%) | 268 (36%) | 113 (32%) |  |
| >75 | 429 (39%) | 290 (39%) | 139 (39%) |  |
| Year of operation |  |  |  |  |
| 2000-2005 | 342 (31%) | 236 (32%) | 106 (30%) | 0.44 |
| 2006-2010 | 353 (32%) | 243 (33%) | 110 (31%) |  |
| 2011-2015 | 405 (37%) | 264 (36%) | 141 (39%) |  |
| Tumor location |  |  |  |  |
| Proximal colon | 536 (49%) | 365 (49%) | 171 (48%) | 0.28 |
| Distal colon | 404 (37%) | 263 (35%) | 141 (39%) |  |
| Rectum | 160 (15%) | 115 (15%) | 45 (13%) |  |
| Disease stage |  |  |  |  |
| I | 184 (17%) | 170 (23%) | 14 (4%) | <0.0001 |
| II | 408 (37%) | 308 (41%) | 100 (28%) |  |
| III | 355 (32%) | 200 (27%) | 155 (43%) |  |
| IV | 153 (14%) | 65 (9%) | 88 (25%) |  |
| Tumour grade |  |  |  |  |
| Low-grade | 882 (80%) | 617 (83%) | 265 (74%) | <0.001 |
| High-grade | 218 (20%) | 126 (17%) | 92 (26%) |  |
| Growth pattern |  |  |  |  |
| Medullary | 21 (2%) | 19 (3%) | 2 (1%) | <0.0001 |
| Micropapillary | 70 (6%) | 23 (3%) | 47 (13%) |  |
| Mucinous | 76 (7%) | 53 (7%) | 23 (6%) |  |
| Signet ring | 28 (3%) | 15 (2%) | 13 (4%) |  |
| Adenocarcinoma NOS | 905 (83%) | 633 (85%) | 272 (76%) |  |
| Lymphovascular invasion |  |  |  |  |
| No | 858 (78%) | 639 (86%) | 219 (61%) | <0.0001 |
| Yes | 242 (22%) | 104 (14%) | 138 (39%) |  |
| Tumor budding |  |  |  |  |
| Bd1 | 827 (75%) | 623 (84%) | 204 (57%) | <0.0001 |
| Bd2 | 156 (14%) | 78 (10%) | 78 (22%) |  |
| Bd3 | 117 (11%) | 42 (6%) | 75 (21%) |  |
| SARIFA status |  |  |  |  |
| Negative | 774 (70%) | 636 (86%) | 138 (39%) | <0.0001 |
| Positive | 326 (30%) | 107 (14%) | 219 (61%) |  |
| MMR status |  |  |  |  |
| Proficient | 931 (85%) | 604 (81%) | 327 (92%) | <0.0001 |
| Deficient | 169 (15%) | 139 (19%) | 30 (8%) |  |
| *BRAF* status^A^ |  |  |  |  |
| Wild-type | 916 (83%) | 608 (82%) | 308 (87%) | 0.056 |
| Mutant | 182 (17%) | 134 (18%) | 48 (13%) |  |

**Table S1.** Patient and tumor characteristics and their associations with tumor-stroma ratio (TSR)

Abbreviations: MMR, Mismatch repair

A Data missing for 2 cases.
