# Supplementary material for: Impact of stromal maturity and proportion on prognosis and immune landscape in colorectal cancer
Source: Ann Med. 2025 Dec 26;58(1):2606512. doi: 10.1080/07853890.2025.2606512 (PMC12777758; doi:10.1080/07853890.2025.2606512)
Supplement: supplementary tables.zip [file IANN_A_2606512_SM3390.zip › TableS2.docx]

**Table S2.** Patient and tumor characteristics and their associations with Desmoplastic reaction (DR) classification

|  |  | DR classification | | |  |
| --- | --- | --- | --- | --- | --- |
| Characteristic | Total N | Mature | Intermediate | Immature | P |
| All cases | 1100 | 613 (56%) | 154 (14%) | 333 (30%) |  |
| Sex |  |  |  |  |  |
| Female | 543 (49%) | 292 (48%) | 80 (52%) | 171 (51%) | 0.43 |
| Male | 557 (51%) | 321 (52%) | 74 (48%) | 162 (49%) |  |
| Age (years) |  |  |  |  |  |
| <65 | 290 (26%) | 156 (25%) | 38 (25%) | 96 (29%) | 0.28 |
| 65-75 | 381 (35%) | 216 (35%) | 46 (30%) | 119 (36%) |  |
| >75 | 429 (39%) | 241 (39%) | 70 (45%) | 118 (35%) |  |
| Year of operation |  |  |  |  |  |
| 2000-2005 | 342 (31%) | 291 (33%) | 45 (29%) | 96 (29%) | 0.54 |
| 2006-2010 | 353 (32%) | 198 (32%) | 46 (30%) | 109 (33%) |  |
| 2011-2015 | 405 (37%) | 214 (35%) | 63 (41%) | 128 (38%) |  |
| Tumor location |  |  |  |  |  |
| Proximal colon | 536 (49%) | 278 (45%) | 93 (60%) | 165 (50%) | 0.003 |
| Distal colon | 404 (37%) | 233 (38%) | 40 (26%) | 131 (39%) |  |
| Rectum | 160 (15%) | 102 (17%) | 21 (14%) | 37 (11%) |  |
| Disease stage |  |  |  |  |  |
| I | 184 (17%) | 147 (24%) | 31 (20%) | 6 (2%) | <0.0001 |
| II | 408 (37%) | 276 (45%) | 57 (37%) | 75 (23%) |  |
| III | 355 (32%) | 144 (23%) | 46 (30%) | 165 (50%) |  |
| IV | 153 (14%) | 46 (8%) | 20 (13%) | 87 (26%) |  |
| Tumour grade |  |  |  |  |  |
| Low-grade | 882 (80%) | 527 (86%) | 113 (73%) | 242 (73%) | <0.0001 |
| High-grade | 218 (20%) | 86 (13%) | 41 (27%) | 91 (27%) |  |
| Growth pattern |  |  |  |  |  |
| Medullary | 21 (2%) | 15 (2%) | 5 (3%) | 1 (0%) | <0.0001 |
| Micropapillary | 70 (6%) | 16 (3%) | 13 (8%) | 41 (12%) |  |
| Mucinous | 76 (7%) | 44 (7%) | 16 (10%) | 16 (5%) |  |
| Signet ring | 28 (3%) | 13 (2%) | 2 (1%) | 13 (4%) |  |
| Adenocarcinoma NOS | 905 (83%) | 525 (85%) | 118 (76%) | 262 (79%) |  |
| Lymphovascular invasion |  |  |  |  |  |
| No | 858 (78%) | 540 (88%) | 122 (79%) | 196 (59%) | <0.0001 |
| Yes | 242 (22%) | 73 (12%) | 32 (21 %) | 137 (41%) |  |
| Tumor budding |  |  |  |  |  |
| Bd1 | 827 (75%) | 541 (88%) | 108 (70%) | 178 (53%) | <0.0001 |
| Bd2 | 156 (14%) | 50 (8%) | 29 (19%) | 77 (35%) |  |
| Bd3 | 117 (11%) | 22 (4%) | 17 (11%) | 78 (23%) |  |
| SARIFA status |  |  |  |  |  |
| Negative | 774 (70%) | 576 (94%) | 103 (67%) | 95 (29%) | <0.0001 |
| Positive | 326 (30%) | 37 (6%) | 51 (33%) | 238 (71%) |  |
| MMR status |  |  |  |  |  |
| Proficient | 931 (85%) | 511 (83%) | 121 (79%) | 299 (90%) | 0.003 |
| Deficient | 169 (15%) | 102 (17%) | 33 (21%) | 34 (10%) |  |
| *BRAF* status^A^ |  |  |  |  |  |
| Wild-type | 916 (83%) | 523 (86%) | 118 (77%) | 275 (83%) | 0.025 |
| Mutant | 182 (17%) | 88 (14%) | 36 (23%) | 58 (17%) |  |

Abbreviations: MMR, Mismatch repair

A Data missing for 2 cases.
