# Supplementary material for: Impact of stromal maturity and proportion on prognosis and immune landscape in colorectal cancer
Source: Ann Med. 2025 Dec 26;58(1):2606512. doi: 10.1080/07853890.2025.2606512 (PMC12777758; doi:10.1080/07853890.2025.2606512)
Supplement: supplementary tables.zip [file IANN_A_2606512_SM3390.zip › TableS3.docx]

**Table S3.** Multivariable Cox regression models for cancer-specific survival

|  | Multivariable HR (95% CI) | | |
| --- | --- | --- | --- |
|  | SMAPS | DR classification | TSR |
| SMAPS |  |  |  |
| Low | 1 (referent) | - | - |
| Intermediate | 1.50 (1.08-2.07) | - | - |
| High | 2.01 (1.47-2.75) | - | - |
| DR classification |  |  |  |
| Mature | - | 1 (referent) | - |
| Intermediate | - | 1.11 (0.75-1.66) | - |
| Immature | - | 1.84 (1.39-2.45) | - |
| TSR |  |  |  |
| Low | - | - | 1 (referent) |
| High | - | - | 1.49 (1.15-1.93) |
| Age |  |  |  |
| <65 | 1 (referent) | 1 (referent) | 1 (referent) |
| 65-75 | 1.10 (0.81-1.47) | 1.08 (0.80-1.45) | 1.13 (0.84-1.52) |
| >75 | 1.81 (1.35-2.43) | 1.80 (1.35-2.41) | 1.81 (1.35-2.42) |
| Gender |  |  |  |
| Male | 1 (referent) | 1 (referent) | 1 (referent) |
| Female | 0.86 (0.67-1.09) | 0.86 (0.68-1.09) | 0.86 (0.67-1.09) |
| Year of operation |  |  |  |
| 2000-2005 | 1 (referent) | 1 (referent) | 1 (referent) |
| 2006-2010 | 0.60 (0.45-0.80) | 0.59 (0.45-0.79) | 0.62 (0.46-0.82) |
| 2011-2015 | 0.47 (0.35-0.63) | 0.47 (0.35-0.63) | 0.49 (0.37-0.65) |
| Tumor location |  |  |  |
| Proximal colon | 1 (referent) | 1 (referent) | 1 (referent) |
| Distal colon | 0.90 (0.69-1.17) | 0.89 (0.68-1.16) | 0.91 (0.70-1.19) |
| Rectum | 0.86 (0.60-1.25) | 0.85 (0.59-1.23) | 0.85 (0.59-1.23) |
| Disease stage |  |  |  |
| I-II | 1 (referent) | 1 (referent) | 1 (referent) |
| III | 2.67 (1.92-3.69) | 2.68 (1.94-3.71) | 2.87 (2.08-3.96) |
| IV | 14.8 (10.4-21.0) | 15.1 (10.6-21.4) | 16.1 (11.4-22.8) |
| Tumor grade |  |  |  |
| Low-grade | 1 (referent) | 1 (referent) | 1 (referent) |
| High-grade | 1.49 (1.12-1.98) | 1.53 (1.15-2.03) | 1.51 (1.13-2.02) |
| Lymphovascular invasion |  |  |  |
| No | 1 (referent) | 1 (referent) | 1 (referent) |
| Yes | 1.53 (1.18-1.99) | 1.58 (1.22-2.04) | 1.61 (1.24-2.10) |
| Tumor budding |  |  |  |
| Bd1 | 1 (referent) | 1 (referent) | 1 (referent) |
| Bd2 | 1.21 (0.89-1.65) | 1.24 (0.91-1.69) | 1.24 (0.91-1.69) |
| Bd3 | 1.18 (0.86-1.63) | 1.20 (0.87-1.65) | 1.25 (0.90-1.72) |
| *BRAF* mutation |  |  |  |
| Wild-type | 1 (referent) | 1 (referent) | 1 (referent) |
| Mutant | 1.36 (0.89-2.07) | 1.31 (0.86-1.99) | 1.38 (0.90-2.11) |
| MMR status |  |  |  |
| Proficient | 1 (referent) | 1 (referent) | 1 (referent) |
| Deficient | 0.71 (0.43-1.19) | 0.70 (0.42-1.16) | 0.67 (0.40-1.11) |

Abbreviations: HR, hazard ratio; CI, confidence interval; MMR, mismatch repair.
