# Supplementary material for: Impact of stromal maturity and proportion on prognosis and immune landscape in colorectal cancer
Source: Ann Med. 2025 Dec 26;58(1):2606512. doi: 10.1080/07853890.2025.2606512 (PMC12777758; doi:10.1080/07853890.2025.2606512)
Supplement: supplementary tables.zip [file IANN_A_2606512_SM3390.zip › TableS4.docx]

**Table S4.** Comparison of the prognostic power of SMAPS and tumor budding using Cox regression models for cancer-specific survival.

| Variable | No. of cases | No. of events | Model 1 (Univariable) HR (95% CI) | Model 2 (multivariable) HR (95% CI) | Model 3 (multivariable) HR (95% CI) |
| --- | --- | --- | --- | --- | --- |
| SMAPS |  |  |  |  |  |
| Low | 630 | 101 | 1 (referent) | 1 (referent) | 1 (referent) |
| Intermediate | 206 | 72 | 2.63 (1.94-3.55) | 2.36 (1.74-3.22) | 1.50 (1.08-2.07) |
| High | 227 | 123 | 4.64 (3.57-6.05) | 3.76 (2.82-5.01) | 2.01 (1.47-2.75) |
| P_trend_ |  |  | <0.0001 | <0.0001 | <0.0001 |
| Tumor budding |  |  |  |  |  |
| Bd1 | 803 | 175 | 1 (referent) | 1 (referent) | 1 (referent) |
| Bd2 | 148 | 61 | 2.24 (1.68-3.00) | 1.50 (1.11-2.04) | 1.21 (0.89-1.65) |
| Bd3 | 112 | 60 | 3.20 (2.39-4.30) | 1.86 (1.36-2.54) | 1.18 (0.86-1.63) |
| P_trend_ |  |  | <0.0001 | <0.0001 | 0.23 |

The analysis included patients from whom both the SMAPS and tumor budding were determined. The patients who had received preoperative treatments or died within 30 days or less after the surgery were excluded, resulting 983 patients.

Model 2: Cox proportional hazards regression model that included SMAPS and tumor budding.

Model 3: Cox proportional hazards regression model that included SMAPS and tumor budding, and was additionally adjusted for age (<65, 65-75, <75), sex (male, female), stage (I-II, III, IV), tumor location (proximal colon, distal colon, rectum), year of operation (2000-2005, 2006-2010, 2011-2015), lymphatic or venous invasion (no, yes), grade (low-grade, high-grade), MMR status (proficient, deficient), and *BRAF* (wild-type, mutant).

P_trend_ values were calculated by using three ordinal categories of SMAPS and tumor budding continuous variables in univariable and multivariable Cox proportional hazard regression models.

Abbreviations: HR, hazard ratio; CI, confidence interval
