# Supplementary material for: Impact of stromal maturity and proportion on prognosis and immune landscape in colorectal cancer
Source: Ann Med. 2025 Dec 26;58(1):2606512. doi: 10.1080/07853890.2025.2606512 (PMC12777758; doi:10.1080/07853890.2025.2606512)
Supplement: supplementary tables.zip [file IANN_A_2606512_SM3390.zip › TableS6.docx]

**Table S6.** The prognostic power of tumor-stroma ratio (TSR), desmoplastic reaction (DR) classification, the Stroma Maturity and Proportion Score (SMAPS), and tumor budding in stage II colorectal cancer patients using Cox regression models for cancer-specific survival.

| Variable | No. of cases | No. of events | Univariable  HR (95% CI) | Multivariable  HR (95% CI) |
| --- | --- | --- | --- | --- |
| **Study cohort** |  |  |  |  |
| TSR |  |  |  |  |
| Stroma-low | 300 | 29 | 1 (referent) | 1 (referent) |
| Stroma-high | 96 | 21 | 2.59 (1.48-4.54) | 2.45 (1.35-4.45) |
| P |  |  | 0.0009 | 0.003 |
| DR classification |  |  |  |  |
| Mature | 269 | 29 | 1 (referent) | 1 (referent) |
| Intermediate | 53 | 5 | 0.88 (0.34-2.28) | 1.06 (0.40-2.82) |
| Immature | 74 | 16 | 2.15 (1.16-3.95) | 2.19 (1.13-4.24) |
| P_trend_ |  |  | 0.024 | 0.026 |
| SMAPS |  |  |  |  |
| Low | 277 | 26 | 1 (referent) | 1 (referent) |
| Intermediate | 68 | 11 | 1.89 (0.94-3.83) | 1.94 (0.93-4.02) |
| High | 51 | 13 | 3.08 (1.58-6.00) | 2.98 (1.44-6.17) |
| P_trend_ |  |  | 0.0006 | 0.002 |
| Tumor budding |  |  |  |  |
| Bd1 | 327 | 32 | 1 (referent) | 1 (referent) |
| Bd2 | 44 | 12 | 3.47 (1.79-6.75) | 3.55 (1.77-7.14) |
| Bd3 | 25 | 6 | 2.63 (1.10-6.30) | 2.38 (0.95-5.94) |
| P_trend_ |  |  | 0.0007 | 0.003 |
| **Validation cohort** |  |  |  |  |
| TSR |  |  |  |  |
| Stroma-low | 156 | 9 | 1 (referent) | 1 (referent) |
| Stroma-high | 97 | 9 | 1.73 (0.69-4.37) | 1.76 (0.66-4.69) |
| P |  |  | 0.24 | 0.26 |
| DR classification |  |  |  |  |
| Mature | 146 | 9 | 1 (referent) | 1 (referent) |
| Intermediate | 39 | 1 | 0.42 (0.053-3.34) | 0.32 (0.038-2.69) |
| Immature | 68 | 8 | 2.58 (0.99-6.75) | 2.76 (0.87-8.79) |
| P_trend_ |  |  | 0.079 | 0.14 |
| SMAPS |  |  |  |  |
| Low | 117 | 7 | 1 (referent) | 1 (referent) |
| Intermediate | 107 | 5 | 0.93 (0.29-2.93) | 1.02 (0.30-3.44) |
| High | 29 | 6 | 4.74 (1.58-14.2) | 5.58 (1.49-21.0) |
| P_trend_ |  |  | 0.022 | 0.029 |
| Tumor budding |  |  |  |  |
| Bd1 | 202 | 13 | 1 (referent) | 1 (referent) |
| Bd2 | 32 | 3 | 1.40 (0.40-4.93) | 1.07 (0.27-4.20) |
| Bd3 | 19 | 2 | 1.59 (0.36-7.05) | 1.03 (0.21-5.12) |
| P_trend_ |  |  | 0.46 | 0.95 |

The analysis included stage II patients from the study cohort and validation cohort. The patients who had received preoperative treatments or died within 30 days or less after the surgery were excluded, resulting 396 patients in study cohort and 253 patients in validation cohort. Multivariable Cox proportional hazards regression model was adjusted for age (<65, 65-75, <75), sex (male, female), tumor location (proximal colon, distal colon, rectum), year of operation (2000-2005, 2006-2010, 2011-2015), lymphatic or venous invasion (no, yes), grade (low-grade, high-grade), MMR status (proficient, deficient), and *BRAF* (wild-type, mutant).

P_trend_ values were calculated by using three ordinal categories of DR classification, SMAPS, and tumor budding as continuous variables in univariable and multivariable Cox proportional hazard regression models.

Abbreviations: HR, hazard ratio; CI, confidence interval
