# Supplementary material for: Impact of stromal maturity and proportion on prognosis and immune landscape in colorectal cancer
Source: Ann Med. 2025 Dec 26;58(1):2606512. doi: 10.1080/07853890.2025.2606512 (PMC12777758; doi:10.1080/07853890.2025.2606512)
Supplement: supplementary tables.zip [file IANN_A_2606512_SM3390.zip › TableS8.docx]

**Table S8**. Patient and tumor characteristics and their associations with Stroma Maturity and Proportion Score (SMAPS) in the validation cohort

|  |  | SMAPS | | |  |
| --- | --- | --- | --- | --- | --- |
| Characteristic | Total N | Low | Intermediate | High | P |
| All cases | 776 | 362 (47%) | 284 (37%) | 130 (17%) |  |
| Sex |  |  |  |  |  |
| Female | 364 (47%) | 180 (50%) | 127 (45%) | 57 (44%) | 0.33 |
| Male | 412 (53%) | 182 (50%) | 157 (55%) | 73 (56%) |  |
| Age (years) |  |  |  |  |  |
| <65 | 233 (30%) | 95 (26%) | 90 (32%) | 48 (37%) | 0.036 |
| 65-75 | 285 (37%) | 129 (36%) | 113 (40%) | 43 (33%) |  |
| >75 | 258 (33%) | 138 (38%) | 81 (29%) | 39 (30%) |  |
| Year of operation |  |  |  |  |  |
| 2006-2010 | 155 (20%) | 79 (22%) | 48 (17%) | 28 (22%) | 0.015 |
| 2011-2015 | 218 (28%) | 113 (31%) | 82 (29%) | 23 (18%) |  |
| 2016-2020 | 403 (52%) | 170 (47%) | 154 (54%) | 79 (61%) |  |
| Tumor location |  |  |  |  |  |
| Proximal colon | 323 (42%) | 162 (45%) | 119 (42%) | 42 (32%) | 0.038 |
| Distal colon | 205 (26%) | 81 (22%) | 78 (27%) | 46 (35%) |  |
| Rectum | 248 (32%) | 119 (33%) | 87 (31%) | 42 (32%) |  |
| Disease stage |  |  |  |  |  |
| I | 187 (24%) | 134 (37%) | 47 (17%) | 6 (5%) | <0.0001 |
| II | 253 (33%) | 117 (32%) | 107 (38%) | 29 (22%) |  |
| III | 251 (32%) | 90 (25%) | 95 (33%) | 66 (51%) |  |
| IV | 85 (11%) | 21 (6%) | 35 (12%) | 29 (22%) |  |
| Tumour grade |  |  |  |  |  |
| Low-grade | 665 (86%) | 317 (88%) | 237 (83%) | 111 (85%) | 0.33 |
| High-grade | 111 (14%) | 45 (12%) | 47 (17%) | 19 (15%) |  |
| Growth pattern |  |  |  |  |  |
| Medullary | 14 (2%) | 6 (2%) | 8 (3%) | 0 (0%) | 0.001 |
| Micropapillary | 38 (5%) | 9 (2%) | 16 (6%) | 13 (10%) |  |
| Mucinous | 62 (8%) | 33 (9%) | 24 (8%) | 6 (4%) |  |
| Signet rign | 15 (2%) | 9 (2%) | 4 (1%) | 2 (2%) |  |
| Adenocarcinoma NOS | 646 (83%) | 305 (84%) | 232 (82%) | 110 (85%) |  |
| Lymphovascular invasion |  |  |  |  |  |
| No | 429 (55%) | 244 (67%) | 150 (53%) | 335 (27%) | <0.0001 |
| Yes | 347 (45%) | 118 (33%) | 134 (47%) | 95 (73%) |  |
| Tumor budding |  |  |  |  |  |
| Bd1 | 541 (70%) | 288 (80%) | 203 (71%) | 50 (38%) | <0.0001 |
| Bd2 | 129 (17%) | 49 (14%) | 47 (17%) | 33 (25%) |  |
| Bd3 | 106 (14%) | 25 (7%) | 34 (12%) | 47 (36%) |  |
| SARIFA status |  |  |  |  |  |
| Negative | 533 (69%) | 312 (86%) | 183 (64%) | 38 (29%) | <0.0001 |
| Positive | 243 (31%) | 50 (14%) | 101 (36 %) | 92 (71%) |  |
| MMR status |  |  |  |  |  |
| Proficient | 652 (84%) | 287 (79%) | 241 (85%) | 124 (95%) | <0.0001 |
| Deficient | 124 (16%) | 75 (21%) | 43 (15%) | 6 (5%) |  |
| *BRAF* status^A^ |  |  |  |  |  |
| Wild-type | 662 (86%) | 297 (83%) | 244 (86%) | 121 (93%) | 0.025 |
| Mutant | 107 (14%) | 59 (17%) | 39 (14%) | 9 (7%) |  |

Abbreviations: MMR, Mismatch repair

A Data missing for 7 cases
