# Supplementary material for: Impact of stromal maturity and proportion on prognosis and immune landscape in colorectal cancer
Source: Ann Med. 2025 Dec 26;58(1):2606512. doi: 10.1080/07853890.2025.2606512 (PMC12777758; doi:10.1080/07853890.2025.2606512)
Supplement: supplementary tables.zip [file IANN_A_2606512_SM3390.zip › TableS9.docx]

**Table S9.** Multivariable Cox regression models for cancer-specific survival in the validation cohort.

| **Variable** | Multivariable HR (95% CI) |
| --- | --- |
| SMAPS |  |
| Low | 1 (referent) |
| Intermediate | 1.34 (0.88-2.04) |
| High | 2.53 (1.58-4.05) |
| Age |  |
| <65 | 1 (referent) |
| 65-75 | 1.76 (1.16-2.66) |
| >75 | 2.97 (1.94-4.54) |
| Sex |  |
| Male | 1 (referent) |
| Female | 1.02 (0.73-1.42) |
| Year of operation |  |
| 2006-2010 | 1 (referent) |
| 2011-2015 | 1.10 (0.72-1.67) |
| 2016-2020 | 0.57 (0.37-0.87) |
| Tumor location |  |
| Proximal colon | 1 (referent) |
| Distal colon | 1.10 (0.73-1.66) |
| Rectum | 1.00 (0.66-1.51) |
| Disease stage |  |
| I-II | 1 (referent) |
| III | 2.41 (1.46-3.98) |
| IV | 18.5 (10.9-31.4) |
| Tumor grade |  |
| Low-grade | 1 (referent) |
| High-grade | 1.39 (0.91-2.10) |
| Lymphovascular invasion |  |
| No | 1 (referent) |
| Yes | 1.67 (1.07-2.59) |
| Tumor budding |  |
| Grade 1 | 1 (referent) |
| Grade 2 | 1.57 (1.02-2.41) |
| Grade 3 | 1.44 (0.93-2.21) |
| *BRAF* mutation |  |
| Wild-type | 1 (referent) |
| Mutant | 1.73 (0.96-3.12) |

Abbreviations: HR, hazard ratio; CI, confidence interval; MMR, mismatch repair; SMAPS, Stroma Maturity and Proportion Score.
