# Supplementary material for: Impact of stromal maturity and proportion on prognosis and immune landscape in colorectal cancer
Source: Ann Med. 2025 Dec 26;58(1):2606512. doi: 10.1080/07853890.2025.2606512 (PMC12777758; doi:10.1080/07853890.2025.2606512)
Supplement: supplementary tables.zip [file IANN_A_2606512_SM3390.zip › TableS10.docx]

**Table S10.** T cells densities according to the Stroma Maturity and Proportion Score (SMAPS) in the validation cohort.

|  |  | **SMAPS** | | |  |
| --- | --- | --- | --- | --- | --- |
| Immune cell type | N | Low | Intermediate | High | P |
| **Tumor center** |  |  |  |  |  |
| CD3+ T cells | 751 | 679 (393-1060) | 651 (342-1050) | 408 (228-663) | <0.0001 |
| CD8+ T cells | 751 | 223 (106-451) | 213 (87.0-428) | 130 (48.6-225) | <0.0001 |
| **Invasive margin** |  |  |  |  |  |
| CD3+ T cells | 751 | 817 (489-1230) | 825 (457-1350) | 552 (257-919) | <0.0001 |
| CD8+ T cells | 751 | 373 (170-648) | 357 (187-685) | 237 (104-452) | <0.0001 |

The numbers indicate T cell densities (1/mm^2^).
